# Design of a Soft Robotic Artificial Cardiac Wall

**DOI:** 10.1111/aor.14978

**Published:** 2025-03-12

**Authors:** Debora Zrinscak, Claudia M. De Chirico, Lucrezia Lorenzon, Fabiola Coluccia, Mauro De Luca, Martina Maselli, Jolanda Kluin, Johannes T. B. Overvelde, Matteo Cianchetti

**Affiliations:** ^1^ The BioRobotics Insitute and Department of Excellence of Robotics & AI, Scuola Superiore Sant'Anna Pontedera Italy; ^2^ Department of Cardiothoracic Surgery Thorax Center, Erasmus MC Rotterdam Rotterdam GD the Netherlands; ^3^ AMOLF Amsterdam the Netherlands

**Keywords:** artificial cardiac wall, cardiovascular disease, heart failure, McKibben actuators, soft robotics

## Abstract

**Background:**

In cardiovascular engineering, the recent introduction of soft robotic technologies sheds new light on the future of implantable cardiac devices, enabling the replication of complex bioinspired architectures and motions. To support human heart function, assistive devices and total artificial hearts have been developed. However, the system's functionality, hemocompatibility, and overall implantability are still open challenges.

**Methods:**

Here, the design of a soft robotic artificial cardiac wall is presented: the action of a bioinspired myocardium of pneumatic McKibben actuators in a double helix is coupled with an engineered passive and deformable endocardial layer made of silicone. The correlation between the helix angle of the actuators and the ejection fraction of the artificial cardiac wall was preliminarily studied with a simplified analytical model. A FEM model was introduced to represent the complex deformation of the endocardial layer during the actuation of the cardiac wall.

**Results:**

Experimental tests report an ejection fraction of 68%, i.e., 77.2 ± 0.4 mL against 90 mmHg, satisfying the minimum physiological requirements and, therefore, proving the concept's functionality.

**Conclusions:**

The conceived device paves the way for a new generation of innovative approaches where engineered bioinspiration might be the key to future artificial cardiac pumps that could support or even substitute the human failing heart.

## Graphical abstract

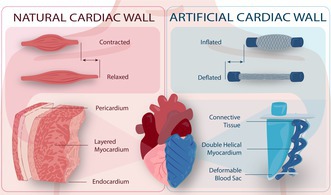

## 1 Introduction

Cardiovascular diseases are among the main causes of death worldwide: 20.5 million people died because of them in 2021 [1, 2]. Genetic predisposition and unhealthy lifestyles [3] are the main risk factors leading to life‐threatening conditions, such as the progressive weakening or stiffening of the cardiac muscle. This long‐term irreversible pathology, known as heart failure, is responsible for inefficient blood pumping functionality, and in end‐stage patients, it can be solved permanently only with a heart transplant [4]. The paucity of donors, however, makes this solution frequently unviable: less than 5000 transplants are performed annually in the US [5]. Moreover, the search for the perfect artificial substitute has been ongoing for nearly a century [6], and a definitive solution is yet to be found.

Nowadays, depending on the patient's condition, ventricular assist devices (VADs) [7] or total artificial hearts (TAHs) [8] are being implanted. VADs usually treat left ventricular heart failure; TAHs, instead, aim to substitute completely both the damaged ventricles [9]. BiVACOR [10], CARMAT Aeson [11], Rein‐Heart [12], and AbioCor [13] are some examples of total artificial heart devices developed in the past few decades. To date, however, the only FDA‐approved bridge‐to‐transplant TAH solution is the SynCardia temporary TAH [14], a pneumatically driven system with percutaneous drivelines. Despite its success, some common complications (e.g., thromboembolism, bleeding, tears, and failures) prevent it from becoming the gold standard treatment for heart failure [15].

The critical challenge in developing a total artificial heart device is not only the satisfaction of mean physiological requirements but also the replication of the complex cardiac shape and motion. Indeed, studies reported a correlation between cardiac structure and functionality in healthy and pathological conditions [16]. Bioinspired designs, aiming at mimicking as closely as possible the natural solutions, became a promising alternative for the development of innovative implantable cardiac devices, minimizing the need to predict the long‐term implications of different design choices, such as providing continuous blood flow. Indeed, the ability to restore a pulsatile blood flow is considered a highly desirable characteristic for an artificial heart [17]; clinical studies on continuous‐flow solutions have reported an increased rate of gastrointestinal bleeding [18] and increased incidence of acquired von Willebrand disease [19].

In the past few decades, soft robotics technologies, based on the use of soft materials and deformable structures, demonstrated the successful replication of complex natural movements for a wide range of purposes [20]. Tasks such as locomotion [21], grasping [22, 23] or swimming [24] are some possible examples. The biomedical field took advantage of this new readily available knowledge and exploited the intrinsic safety and compliance of soft materials to develop surgical manipulators [25], assistive devices [26], and phantoms [27]. Within the cardiovascular field, soft robotics played an important role in the development of both passive phantoms [28], replicating peculiar material properties [29], or structures [30], and in the design of both implantable and non‐implantable systems characterized by embedded actuation. In the latter case, smart active materials were engineered to manufacture cardiac simulators [31, 32, 33] and direct cardiac compression devices [34, 35]. Besides the sole use of deformable bodies, research groups tried to mimic the shape [36, 37, 38], the twist [39, 40], but also the overall myocardial disposition [41], and fiber orientation and motion [42]. The results confirmed how enhanced biomimicry could represent a promising design choice for the development of implantable cardiac devices [43].

This study presents the design of a soft robotic left ventricular wall that integrates the dynamic capabilities of active soft robotics with the beneficial passive properties of materials. Drawing inspiration from natural biomechanics, the double‐helical artificial myocardium induces inward deformation within the functional yet passive endocardial layer. This synergy produces a constrictive motion that mirrors physiological volume reduction, offering a novel actuation methodology that could contribute to the advancement of soft cardiac devices.

## 2 Concept

**FIGURE 1:**
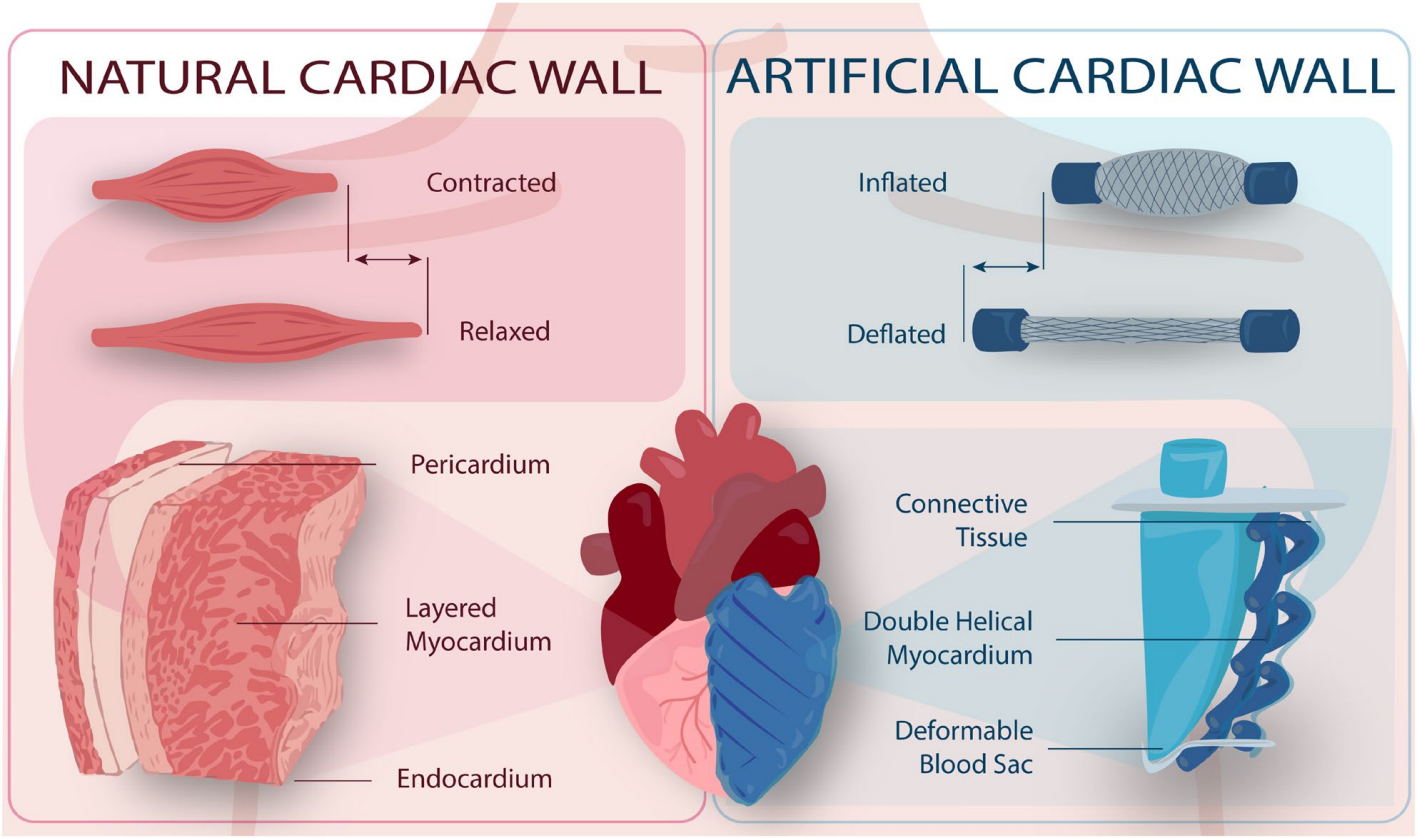
Comparison between the natural (on the left) and the artificial cardiac wall (on the right). Taking inspiration from nature, it was possible to design a bioinspired double‐helical myocardial layer coupled with a deformable endocardium. Upon muscular activation, the endocardial layer deforms, enabling the artificial pumping functionality. [Color figure can be viewed at wileyonlinelibrary.com]

Nature provides a vast array of solutions to various challenges, inspiring robotic design. However, translating natural principles into artificial systems remains challenging [44]. In the natural cardiac wall, the myocardium is contractile, characterized by counteracting helically oriented muscular fibers in clockwise and anti‐clockwise directions [45], with average angles of ±60° reported [45, 46, 47]. Additionally, alterations in ventricular cavity shape, particularly deviations from the semi‐elliptical geometry, often indicate pathological conditions [48]. To replicate the cardiac wall artificially, we abstracted key functional features: (i) a semi‐elliptical endocardial geometry, (ii) two layers of counteracting helical fibers, and (iii) muscle‐like behavior mimicking myocardial contraction. Figure 1 illustrates the translation of these natural features into the artificial design. To optimize contraction during systole, unlike previous approaches embedding muscles in thick silicone structures [43], our concept incorporates an engineered, passive endocardial layer deformed by a double‐helical myocardium. This synergy improved the overall efficiency of the system, enabling the achievement of physiologically relevant ejection fractions with lower energy requirements. The design aimed to replicate physiological pressure (90 mmHg) and achieve an ejection fraction of 50%–70% during the systolic phase. A video summarizing the study's motivation, design, and achievements is available as (Video S1).

## 3 Methods

### 3.1 The Artificial Muscular Unit: The McKibben Actuator

McKibben actuators were studied as basic actuating unit, given their capability of mimicking the natural muscular deformation by coupling axial contraction with radial expansion. Their functionality relies on an inflatable chamber enclosed by an external braided mesh of intertwined threads. When the mesh angle $\theta <54.7^{{}^{\circ}}$, the actuator contracts upon pressurization, achieving a theoretical maximum contraction of 30% of its initial length [49]. Although these actuators have been extensively studied for modeling [50] and sensorization [51], limited research has explored the impact of braided sleeve materials on performance [52]. To develop a low‐pressure, high‐force actuator, multiple mesh materials (polyester, aramid, carbon, innegra, and silane) were tested with Ecoflex 00–30 (Smooth‐On Inc., Macungie, PA) internal chambers. A thin layer of silicone (Ecoflex 00–30 or Dragon Skin 10) was applied to replicate the effects of connective tissue. The manufacturing and experimental testing details of the artificial muscle units are available in the Appendix S1.

### 3.2 Myocardial Layer Analytical Design

A geometrical model was elaborated to define the most promising myocardial actuating unit angle. With this aim, the theoretical contraction of a helically oriented artificial muscle was correlated to the overall volumetric reduction of the ventricular chamber [53]. The main hypotheses on which these calculations were based are the following: (i) both the initial and the final volumes can be simplified as semi‐ellipsoids; (ii) the artificial muscle contracts 20% of its length, in the range of myocardial fibers [54]; (iii) the initial pitch angle of the helix influences the final semi‐major axis dimension; (iv) the final muscular diameter influences the final semi‐minor axis dimension. Defining A as the semi‐major axis and B as the semi‐minor axis, the volume of a semi‐ellipsoid can be defined as:

(1) $$V=\frac{2}{3}\pi {\mathrm{AB}}^{2}$$

And can be generally defined as:

(2) $$\begin{array}{c} f\left(\xi ,\theta \right)=\left\{\begin{array}{c} x=B\;\sin\left(\xi \right)\;\cos\left(\theta \right)\\ y=B\;\sin\left(\xi \right)\;\sin\left(\theta \right)\\ z=A\;\cos\left(\xi \right) \end{array}\right.\;\text{with}\;0\leq \theta \leq 2\pi \;\wedge \;0\leq \xi \leq \frac{\pi }{2} \end{array}$$

Similarly, a general ellipsoidal helix can be derived as:

(3) $$\begin{array}{c} h\left(t\right)=\left\{\begin{array}{c} x=B\;\sin\left(t\right)\;\cos\left(\Omega t\right)\\ y=B\;\sin\left(t\right)\;\sin\left(\Omega t\right)\\ z=A\;\cos\left(t\right) \end{array}\right.\;\text{with}\;\Omega =4n\;\wedge \;0\leq t\leq \frac{\pi }{2} \end{array}$$

where Ω is related to the number of turns n. The length of the actuator can be defined as follows:

(4) $$L=\int_{a}^{b}\sqrt{{\left(\frac{\mathrm{dx}}{\mathrm{dt}}\right)}^{2}+{\left(\frac{\mathrm{dy}}{\mathrm{dt}}\right)}^{2}+{\left(\frac{\mathrm{dz}}{\mathrm{dt}}\right)}^{2}}\mathrm{dt}\;\text{with}\;a\leq t\leq b$$

where a and b are the initial and final points of the three‐dimensional curve. Combining Equations (3) and (4), the general description of the length of an ellipsoidal helix is derived:

(5) $$\begin{array}{c} L=\int_{0}^{\frac{\pi }{2}}\sqrt{{\left(B\;\sin\left(t\right)\right)}^{2}+\left(A+B^{2}\Omega^{2}\right){\left(\cos\left(t\right)\right)}^{2}}\mathrm{dt} \end{array}$$

The pitch angle of the helix α can be defined as:

(6) $$\alpha \left(z\right)=\arccos\left(\sqrt{\frac{\Omega^{2}\left(A^{2}-z^{2}\right)}{\left[\Omega^{2}+{\left(\frac{A}{B}\right)}^{2}\right]\left(A^{2}-z^{2}\right)+z^{2}}}\right)$$

Considering that the contraction of the helix of a parameter k=0.8 is performed with a constant pitch. The variation of semi‐major axis A can be obtained as:

(7) $$\Delta A=A_{i}-A_{f}=z^{*}$$

where i and f, refer to the initial (diastolic) and final (systolic) states, and $z^{*}$ indicates the height of the circumference corresponding to shortened helical fiber. The final length of the helix, $L_{f}={\mathrm{kL}}_{i}$, can also be defined as follows:

(8) $$\begin{array}{c} L_{f}=\int_{0}^{t^{*}}\sqrt{{\left(B_{f}\;\sin\;t\right)}^{2}+\left(A_{f}+B_{f}^{2}\Omega^{2}\right)\left(\cos^{2}\;t\right)}\mathrm{dt} \end{array}$$

where $t^{*}$ is the angle corresponding to $z^{*}$.The only unknown $B_{f}$ was evaluated with a numerical solver. The value was corrected considering that the helical fiber expands radially of a value p upon shortening, as described by the angle θ of the mesh [49]:

(9) $$\left(\begin{array}{c} k=\frac{\cos\;\theta_{f}}{\cos\;\theta_{i}}\\ p=\frac{\sin\;\theta_{f}}{\sin\;\theta_{i}} \end{array}\right.$$

Given all the parameters describing both the initial and final volume of the semi‐ellipsoid, the ideal ejection fraction $\%\mathrm{EF}=\frac{V_{i}-V_{f}}{V_{i}}\%$ was correlated to the pitch angle α and the number of turns n. It is worth mentioning that the initial hypotheses strongly simplify the here‐described system, however, they represented a helpful design starting point.

### 3.3 FEM‐Driven Endocardial Design

To optimize the radial constrictive force during systole, the motion of the active myocardial double‐helical layer was coupled with the action of a passive deformable endocardium. The active and passive layers were connected solely at the apex and base, allowing muscle pressurization to deform the endocardial layer, forming folds and enhancing volumetric reduction, thus enhancing the pumping capability. The material selection had to balance compliance to enable deformation and strength to prevent ballooning under physiological pressures (90 mmHg). Nonlinear static structural FEM simulations (ANSYS Workbench 2021 R1) were run to evaluate three potential artificial endocardium materials, with varying mechanical properties and thicknesses: Ecoflex 00–30 (30 Shore 00, 3 mm), Dragon Skin 30 (30 Shore A, 3 mm), and Smooth‐Sil 950 (50 Shore A, 1.5 mm).

The ventricular chamber was modeled as a semi‐ellipsoidal shell with semi‐axes matching physiological dimensions. Each myocardial layer was represented by four McKibben actuators, modeled as cylindrical bodies with a diameter of 7 mm and defined by helical coordinates in MATLAB. Actuators were divided into 20 segments, of an equal number of points, and each was assigned a local coordinate system for accurate deformation simulation. The chamber and myocardium layers were spaced 1 mm apart.

**FIGURE 2:**
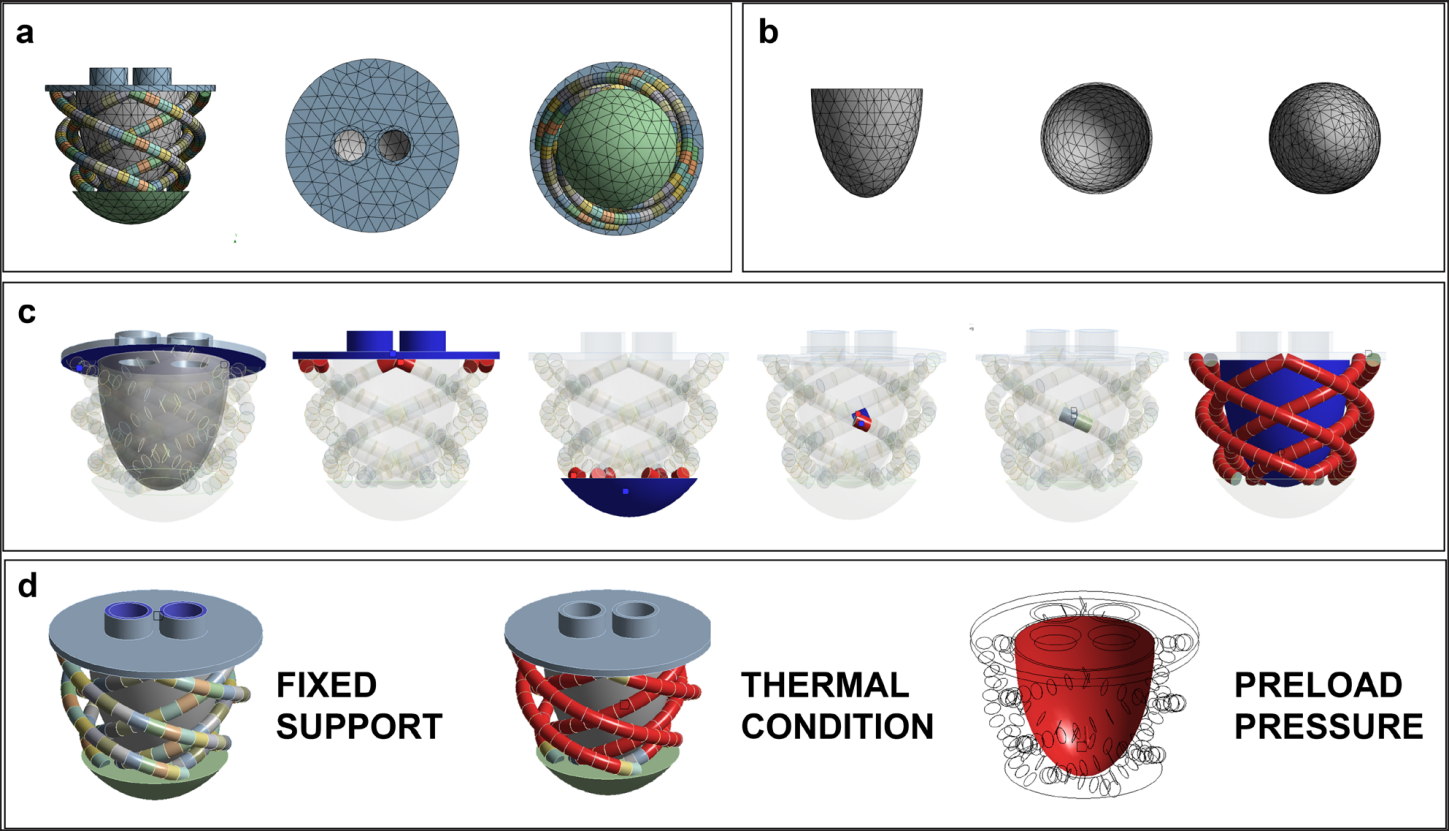
FEM‐guided design of the endocardial layer. (a) Visualization of the mesh applied to the entire model. (b) Detailed view of the mesh on the ventricular region. (c) Contact definitions used in the FEM analysis, with the circle indicating a bonded contact and the star representing a frictional contact. (d) Boundary conditions implemented in the FEM simulation. [Color figure can be viewed at wileyonlinelibrary.com]

Simulations employed quadratic tetrahedral meshing with an average element size of 8.298 mm for the Ecoflex 00–30 and Dragon Skin 30 models, and 8.263 mm for the Smooth‐Sil 950 model, reflecting the reduced thickness of the latter's endocardium. Figure 2a,b illustrates the meshing applied to the entire model and the specific meshing used for the endocardium.

Contact conditions, as shown in Figure 2c, included bonded contacts between rigid plates and both actuator segments and endocardium at apex‐base connections, as well as bonded contacts at the crossing of the inner and outer myocardial layers. Frictional contacts (coefficient: 0.2) were defined between the endocardium and the myocardium layers.

The material properties of the endocardium elastomers were obtained by uniaxial tensile tests on a universal testing machine, following ASTM and ISO standards. As in [40], McKibben actuator properties were modeled as isotropic elastic materials with a Poisson ratio of 0.35 [55] and an experimentally determined Young's modulus of 3.99 MPa. Thermal expansion coefficients were determined experimentally by translating McKibben actuator radial and axial strains under unloaded and loaded conditions into radial and axial expansion coefficients. ABS properties of the plates were sourced from the ANSYS material library, with a thermal expansion coefficient of 1.84 × 10^−4^°C^−1^.

Boundary conditions were set as in (Figure 2d): (i) fixed supports on the upper valve rings constrained chamber motion, (ii) thermal loads applied to actuator segments (excluding the first and last two for stability), and (iii) a pressure of 90 mmHg applied to the endocardial surface, representing peak physiological conditions. Large deflection mode was activated to account for the geometric nonlinearities present in the model. Due to the model instabilities, a stabilization parameter was used to help the convergence, introducing a constant energy dissipation ratio equal to 1. The following load steps were simulated: (i) endocardium pressurization (90 mmHg), and (ii) activation of thermal conditions for muscle contraction. Simulations were performed for actuating pressures ranging from 20 kPa to 120 kPa in 20 kPa increments. The final deformed geometries were exported as STL files via Solidworks (Dassault Systèmes) for volumetric analysis. Experimental validation followed protocol P2 at 90 mmHg, as described in the following section. Additional details on materials and settings are available as Appendix S1.

### 3.4 Artificial Cardiac Wall Fabrication and Testing Protocols

The complete artificial cardiac wall prototypes were fabricated and tested by assembling the active myocardial layers with the passive, deformable endocardium.

#### 3.4.1 Fabrication Procedure

**FIGURE 3:**
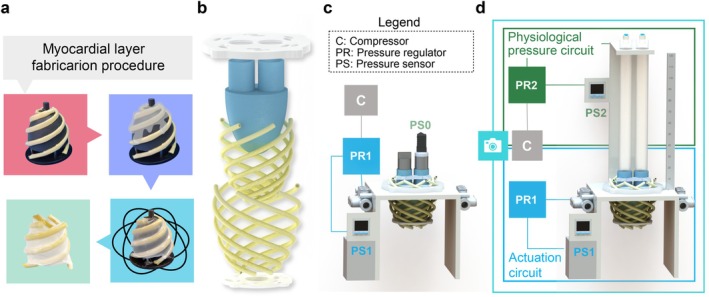
Design steps of a bioinspired myocardial layer and experimental setups. (a) Manufacturing procedure of each myocardial layer: After positioning each actuator on the mold, a thin silicone layer is poured on top of it to secure the position. The mold is rotated until the end of the silicone pot life (45 min); after curing, the myocardial layer is detached from the mold and ready to be assembled. (b) Rendering of the prototype assembly procedure. (c, d) Isolvolumetric test and ejection phase test setup schematics. The abbreviations refer respectively to “PR” pressure regulator, “PS” pressure sensor, and “C” compressed air source. The numbering differentiates the pressure circuits, “0” ventricular pressure circuit, “1” actuation circuit, and “2” physiological pressure simulator circuit. [Color figure can be viewed at wileyonlinelibrary.com]

The artificial double‐helical myocardium was constructed by placing roll‐coated actuators in a custom semi‐ellipsoidal mold featuring helical grooves for precise positioning. Ecoflex 00–30 silicone was poured onto the mold, which was rotated to create a uniform connective tissue layer, as shown in Figure 3a.

The endocardium was fabricated by casting, employing three different materials and two thicknesses: Ecoflex 00–30, Dragon Skin 30 (Smooth‐on Inc., 3 mm thickness) and Smooth‐Sil 950 (Smooth‐on Inc., 1,5 mm). 3D‐printed top and bottom plates facilitated the interface between the endocardium and myocardium. Two prototype configurations were developed: one with four actuators per layer, as modeled in the FEM study, and another with six actuators per layer. Alternative configurations were not considered, as fewer actuators would inadequately cover the endocardial surface. At the same time, a greater number would result in inefficient actuation due to mutual interference between adjacent actuators during pressurization. Additional fabrication details and mold designs are provided as Appendix S1.

#### 3.4.2 Experimental Setups and Protocols

Experimental tests were conducted to validate the FEM simulations and investigate how the number of actuators affects ventricular pressure generation and ejection fraction performance. The experimental performances were compared separately in two phases: the isovolumetric phase and the ejection phase. The respective setups are represented in (Figure 3c,d).

To better analyze the effect of fold formation on system performances the ejection phase was evaluated with two protocols. The tests did not replicate physiological conditions because they lacked valves; however, they provided valuable insights into the expected ejection fraction under dynamic conditions, establishing the upper and lower bounds, respectively. The first protocol (P1), referred to as the “best working condition,” consisted of activating the muscular layer to form folds, leaving the endocardium to deform freely, followed by the application of pressure to the ventricle (afterload, i.e., pressure applied after myocardial contraction). The second protocol (P2), referred to as the “worst working condition,” involved applying static pressure (preload, i.e., pressure applied before muscular activation), followed by the pressurization of the active elements. The expelled volume was evaluated in both cases by observing the water column level.

For the evaluating the ejection phase, two fluidic circuits were employed: one for pressurizing the actuators and the other for simulating physiological pressures within the soft robotic cardiac wall. The first circuit comprised an electronic pressure regulator ITV1050‐21F1BN (SMC, Tokyo, Japan), managing pressures from 0 to 0.99 MPa, and a pressure sensor SWCN‐P10‐P3‐2 (CAMOZZI, Brescia, Italy), with a range of 0 to 1 MPa. The second circuit included a pressure regulator K8P‐0‐E522‐0 (CAMOZZI, Brescia, Italy), managing pressures from 0 to 300 kPa, and a higher‐resolution pressure sensor SWCN‐V01‐P3‐2 (CAMOZZI, Brescia, Italy), managing pressures from −100 to 100 kPa.

The stroke volume was evaluated by video recording the experiments and analyzing the data with motion analysis software (Tracker, physlets.org). Before starting each test, the ventricle was overfilled with an additional 50 mL of water to ensure the water column remained visible when loading was applied. The influence of the water column on ventricular pressure was not accounted for; however, 50 mL corresponds to approximately 12 cm of water, or nearly 9 mmHg, which slightly underestimated the stroke volumes at higher actuation pressures. The relative stroke volume was defined as the difference between the measured volume (water column level) and the design volume (113 mL), whereas the absolute stroke volume was defined as the measured volume subtracted from the maximum volume within the ventricle (with loading applied, end‐diastolic volume). Data were analyzed in the MATLAB environment.

In the first protocol (P1), the actuators were pressurized to a pressure P, the ventricle was inflated in steps of 0.5 kPa from 0.7 kPa to 16.7 kPa (3 mmHg to 120 mmHg), the stroke volume was measured, the pressure of the actuators was incremented, and the test was repeated. In the second protocol (P2), the physiological pressure simulator circuit was set to a specific pressure (evaluated pressures were 0, 30, 60, 90, and 120 mmHg), the actuators were pressurized in steps of 3 kPa, from 15 kPa to 120 kPa and the stroke volume was measured. The physiological pressure was then incremented, and the test was repeated. In both protocols, three prototypes were employed, and each test was repeated three times.

For evaluating the isovolumetric phase, the actuating circuit pressurized the muscles in steps of 3 kPa, from 5 kPa to 110 kPa. The pressure within the ventricle was monitored using a fluidic pressure sensor (RS Pro IPS 797–5043, 0–100 kPa). Three prototypes were employed, with each sample tested five times per test typology. Additionally, to study the loaded behavior of the actuators, the axial and radial strains of the external actuators were measured during the worst working condition. Two actuators per prototype were tested, with each test repeated three times. Axial length was measured by attaching a cotton thread to the base of the actuators and retrieving its length at each pressurization step (from 20 kPa to 120 kPa in steps of 20 kPa). Diameter was measured with a caliper at the aforementioned pressure levels.

Videos of the experimental tests are available as (Video S2).

## 4 Results

### 4.1 Selection of the McKibben Actuator

**FIGURE 4:**
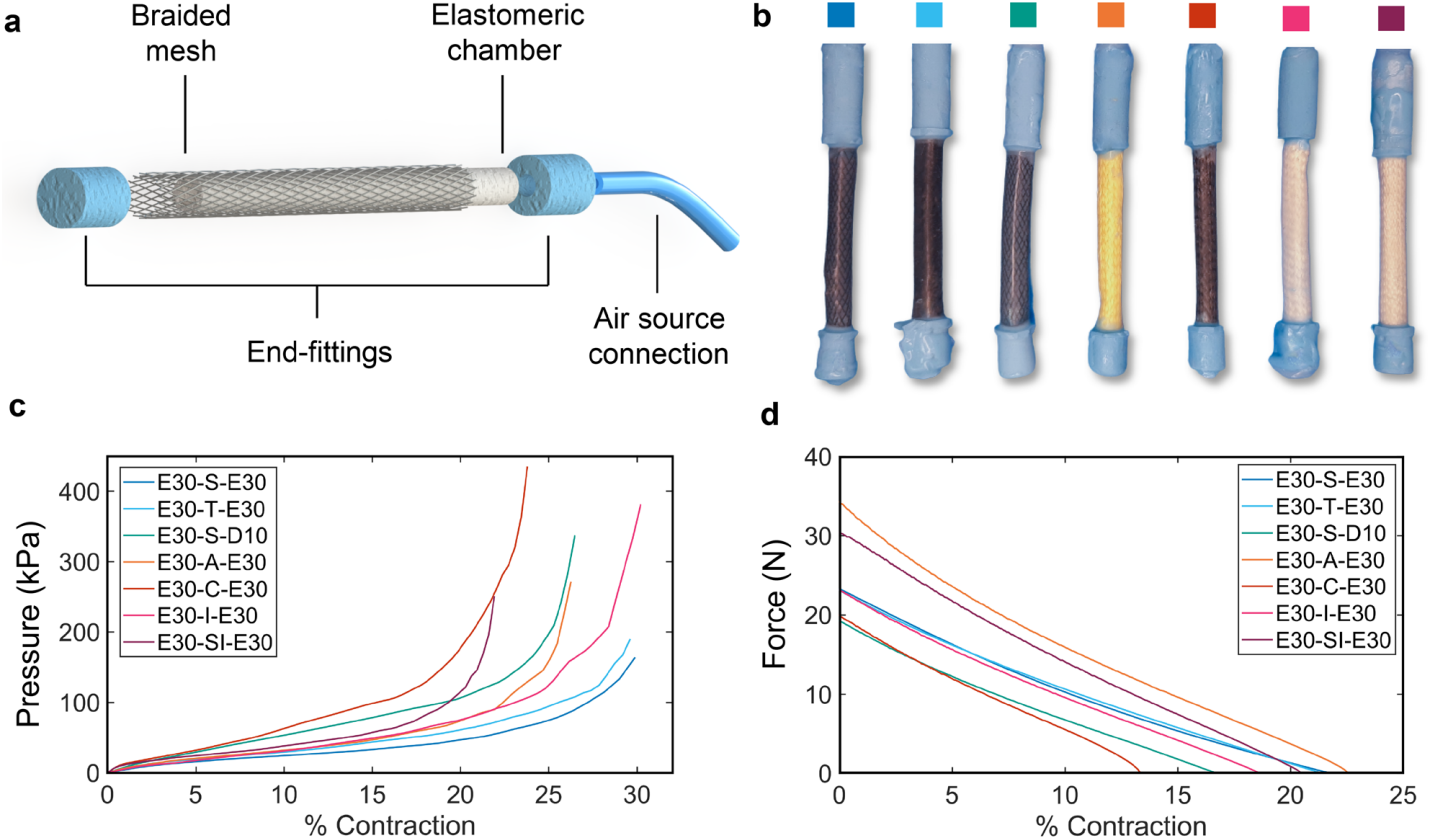
The artificial cardiac muscle. (a) Composition of a McKibben artificial muscle: An inflatable chamber is coupled with a guiding external braided sleeve through two end fittings. One is sealed, and the other connects the actuator to the fluidic line. (b) Investigated prototypes: Inflatable chamber in Ecoflex 00–30, and end fittings in Smooth‐Sil 950. From left to right, the braided sleeve materials are: Polyester (single thread, “S”), polyester (triple thread, “T”), polyester (single thread), aramid (kevlar) “A”, carbon “C”, innegra “I”, and silane “SI”. All muscles were coated with a thin layer of Ecoflex 00–30 (“E30”), exception made for the third prototype coated with Dragon Skin 10 (“D10”). (c) Pressure‐displacement results of the tested McKibben muscles: The maximum contraction ranges from 20% to 30%. The name of each sample is organized as follows: elastomeric chamber material—sleeving material—coating material. (d) Force‐displacement test results for all samples pressurized at 100 kPa. The graph underlines that an aramid braided sleeve allows reaching higher contraction forces at 20% contraction. [Color figure can be viewed at wileyonlinelibrary.com]

All manufactured samples (Figure 4a,b) were characterized to link input pressure with unloaded contraction and force, as shown in Figure 4c,d. Higher actuating pressures generally correspond to larger contraction ratios, forces, and stiffness values. Samples with polyester sleeves (E30‐S‐E30, E30‐T‐E30, E30‐S‐D10) exhibited delamination between the coating and sleeving material and were excluded from further analysis. Carbon (E30‐C‐E30) and silane (E30‐SI‐E30) samples showed lower contraction values but higher bursting pressures, whereas aramid (E30‐A‐E30) and innegra (E30‐I‐E30) actuators demonstrated medium contraction ranges with minimal delamination issues. Figure 4d reports force output at a constant supply pressure of 100 kPa, highlighting material‐dependent stiffness variations. Aramid actuators delivered the highest forces both at rest length and at 20% contraction, making them the most reliable material for low‐pressure, high‐force applications.

### 4.2 Double‐Helical Myocardial Layer Design Results

**FIGURE 5:**
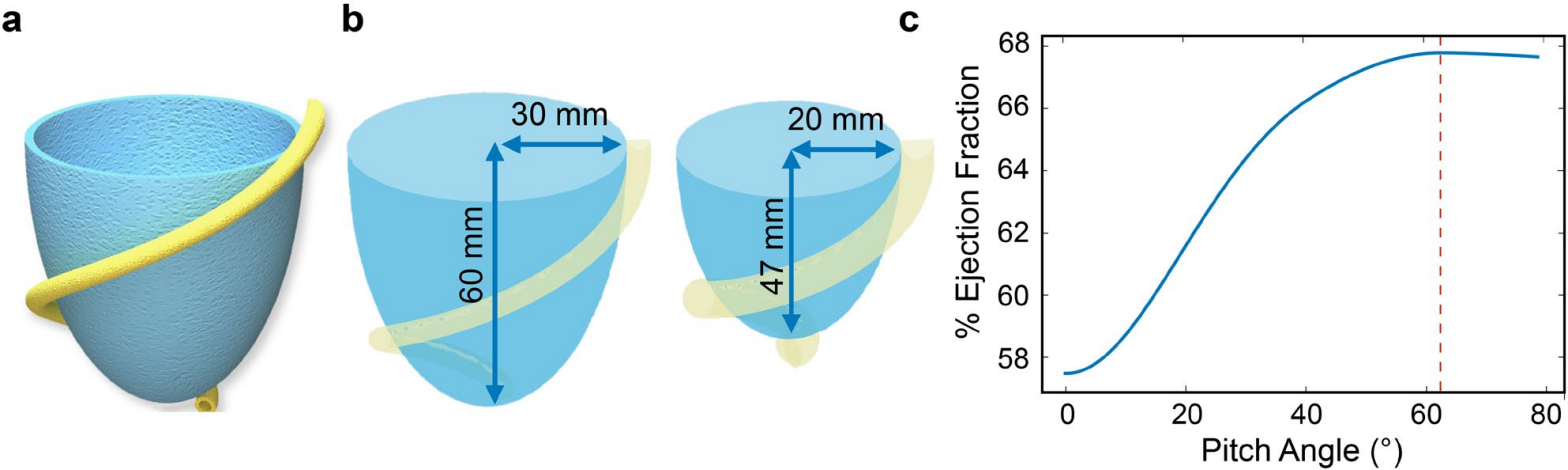
Analytical model results. (a) Rendering of the modeled ventricular chamber surrounded by a McKibben actuator (b) Modeled ventricular deformation, diastolic state with a deflated muscle and systole with a pressurized actuator. The systolic deformed state is modeled as an ideal semi‐ellipsoid. (c) Relation between ejection fraction and pitch angle obtained by modeling the natural ventricle. After an initial and almost linearly increasing section, the curve reaches a plateau corresponding to one helix turn around the chamber. [Color figure can be viewed at wileyonlinelibrary.com]

The ventricular chamber was analytically modeled to determine the optimal orientation of the artificial myocardial fibers. By approximating the natural diastolic geometry as a semi‐ellipsoid with semi‐minor and semi‐major axes of 30 mm and 60 mm, respectively (Figure 5a,b), an end‐diastolic volume of 113 mL was calculated, consistent with physiological values observed in men [56]. Considering this initial volume, the corresponding dimensions, and assuming a 20% contraction of the actuator, the model showed a correlation between the initial actuator angle and the resulting ejection fraction. The highest ejection fraction (68%) seems to correspond to an initial angle of ±63°, falling within the physiologically acceptable range (50%–75%), as shown in Figure 5c. Despite the high level of simplification, given the similarity of the resulting angle compared to the natural myocardial design ($\pm 63^{{}^{\circ}}$ vs. ±60°), we manufactured the prototypes of the two myocardial layers following the analytical model results.

### 4.3 FEM‐Driven Endocardium Selection Results

**FIGURE 6:**
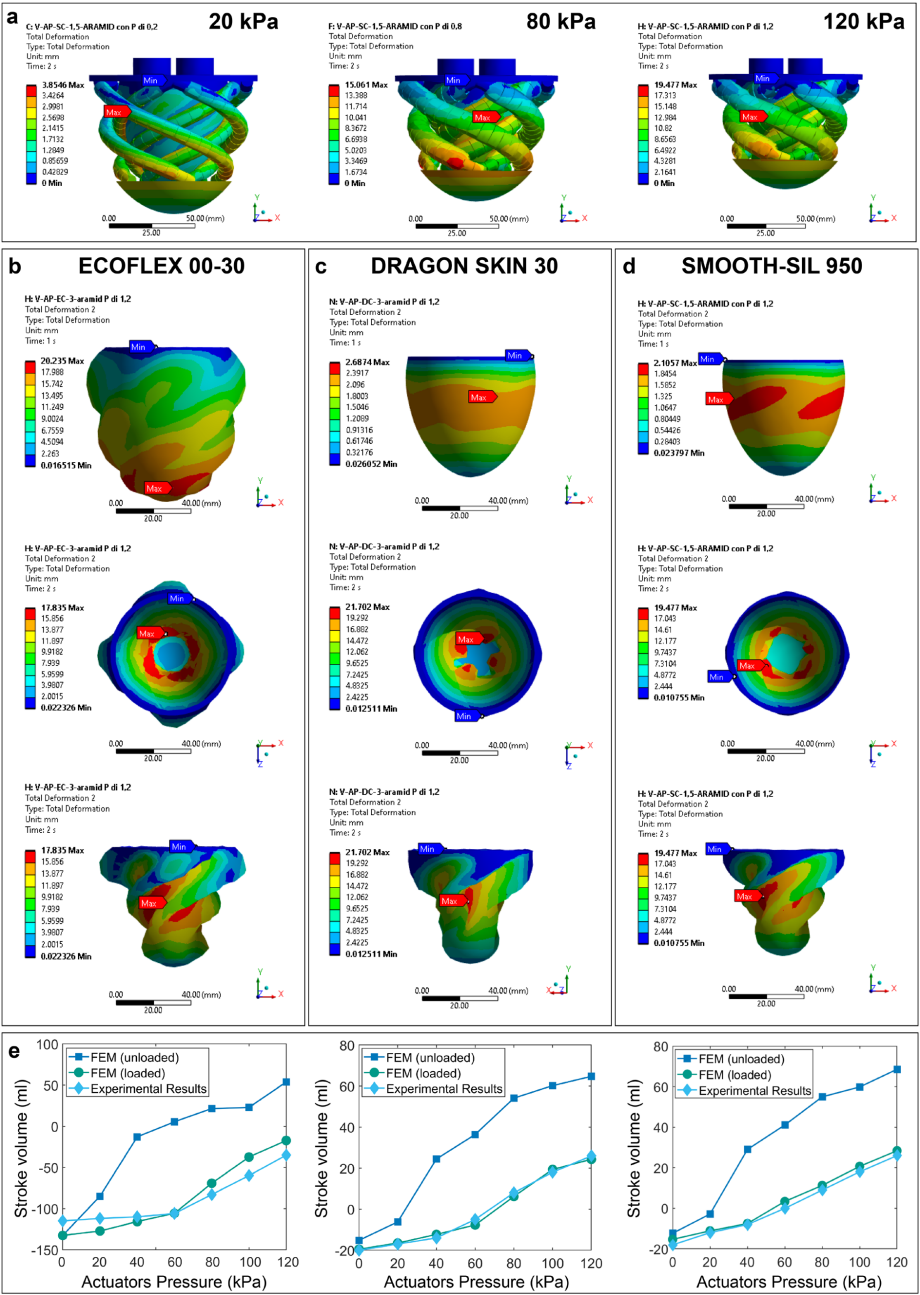
FEM‐guided endocardial layer design results. (a) Contraction dynamics evaluated with FEM studies implementing the McKibben loaded behavior. Shown actuating pressures are 20, 80, and 120 kPa. From (d) to (b–d) endocardial deformation after the preload application, and successively to the activation of the actuators at 120 kPa front and top view, respectively, of Ecoflex 00–30, Dragon Skin 30, Smooth‐Sil 950 endocardia. (e) Diagrams illustrating the comparison between FEM simulation results—representing the behavior of both loaded and unloaded actuators—and the results of experimental validation tests, respectively referred from left to right Ecoflex 00–30, Dragon Skin 30, Smooth‐Sil 950. [Color figure can be viewed at wileyonlinelibrary.com]

The FEM simulations captured the deformation evolution depicted in Figure 6a, modeling different actuator pressurization levels (20, 80, and 120 kPa). The mesh statistics for all models demonstrated good quality, with a minimal percentage of cells flagged for warnings and no cells showing failure issues (see Appendix S1). The deformation of the endocardial layer during the P2 protocol phases—(i) preload application and (ii) myocardial layer activation at 120 kPa—is shown in Figure 6b–d. During muscular activation, the deformed shape of the endocardial layer depends on both its material stiffness and thickness, in some cases creating longitudinal folds that enhance volumetric reduction, as visible in Figure 6c.

Separate FEM studies were performed to analyze the unloaded and loaded behavior of the actuators and were compared with experimental results, as visible in Figure 6e. Experimental data were collected from three P2 tests in the same 8‐actuator prototype typology, and minimal variability was observed between test repetitions. The diagrams show that the unloaded actuators behavior, as expected, significantly overestimated experimental results in all cases but still provided valuable insights into deformation dynamics, particularly fold creation. When comparing FEM results of loaded muscle behavior with experimental tests, varying levels of error were observed. The Dragon Skin 30 and Smooth‐Sil 950 samples achieved maximum RMSE values of 1.6 mL and 2.3 mL, respectively, closely matching the predicted performance. However, the Ecoflex 00–30 sample had an RMSE of 15.1 mL, likely due to abnormal deformation (ballooning) under a physiologically relevant preload pressure of 90 mmHg.

Interestingly, the relative volume reduction in the Smooth‐Sil 950 model (EF 61%) was 16% higher than the Dragon Skin 30 case (EF 56%) and 30% higher than the Ecoflex 00–30 case (EF 47%). The Ecoflex 00–30 chamber was excluded from further analysis due to its low EF values and large volumetric deformation under preload pressure.

To assess whether the overall strain in the models met requirements, maximum normal elastic axial and radial strains were evaluated, as summarized in Table 1. The medium‐soft material (Smooth‐Sil 950) emerged as the most promising sample. It demonstrated similar deformation energy to the softer silicone sample (3.36 J for Smooth‐Sil 950 vs. 3.42 J for Dragon Skin 30) while achieving lower maximum strains.

**TABLE 1:** Maximum normal elastic axial and radial strains.

Material	Maximum normal elastic axial strain [%]	Maximum normal elastic radial strain [%]
Ecoflex 00–30	66.6	41.7
Dragon Skin 30	10.0	17.2
Smooth‐Sil 950	8.6	12.8

### 4.4 Experimental Prototypes and Performances

Experimentally, the influence of the number of actuators was correlated with ventricular pressure generation and ejection fraction performances. Given the designed myocardial arrangement and the selected endocardial material, 8‐actuator and 12‐actuator prototypes, shown in Figure 4a,b, were developed for testing. To define the most promising prototype, isovolumic, and ejection tests (P2) were performed. In both cases, the 12‐actuator prototypes performed better at lower actuation pressures than the 8‐actuator model: generating a higher internal pressure in the first case and ejecting more volume in the second one, Figure 4c,d. The isovolumetric phase was evaluated to determine the correlation between the actuating pressure and natural valves opening. For the 8‐actuator prototype, Figure 4c, the opening of the valves would occur at a mean actuation pressure of 90 kPa, whereas for the 12‐actuator sample, at 80 kPa. This difference also matched the ejection phase evaluations, where a higher stroke volume was reached despite lower actuation pressures in the case of the 12‐actuator prototype, Figure 4d. Being the latter the most promising prototype, further investigations related to the ejection phase were performed.

**FIGURE 7:**
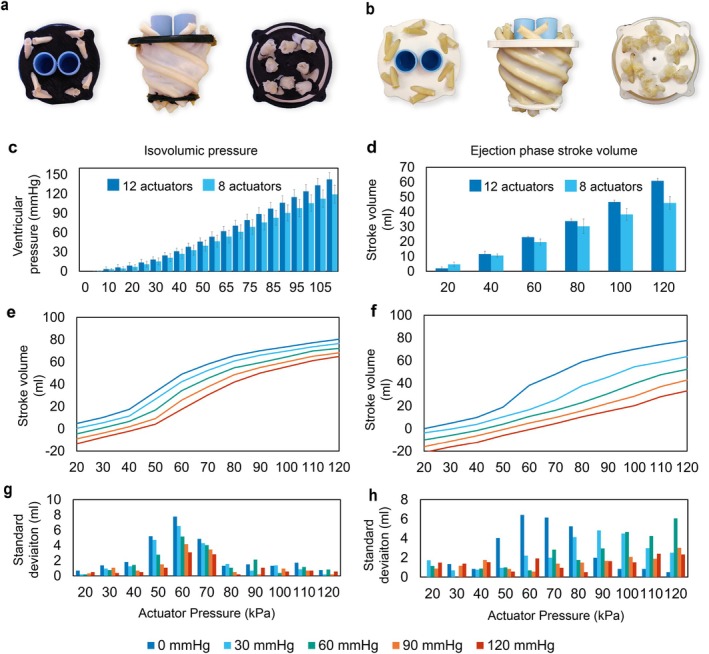
Experimental prototypes and main results. (a) 8‐actuator prototype top, front and bottom view (b) 12‐actuator prototype top, front and bottom view. (c) Isovolumetric phase results, comparison between 8‐actuator and 12‐actuator prototypes, (d) Ejection phase (P2 protocol) absolute stroke volume comparison between 8‐actuator and 12‐actuator prototypes. (e, f) Results of the “best working condition” and “worst working condition”, respectively, at different ventricular pressures. The graphs display the 12‐actuator relative stroke volumes to better underline the trend at different loading pressures. (g, h) Standard deviation behavior corresponding to the above tests. [Color figure can be viewed at wileyonlinelibrary.com] [Color figure can be viewed at wileyonlinelibrary.com]

As visible in Figure 7e, the “best working condition” (P1) results clearly underline the non‐linear behavior of the designed soft robotic cardiac wall. The non‐linearity not only depends on the use of elastomeric materials but also on the formation of inner folds, which are able to form freely in absence of a preload. Figure 7g shows the behavior of the standard deviation. The peak value corresponds to the average actuation pressure triggering the beginning of the formation of longitudinal folds that, depending on the prototype sample and manufacturing differences, are set at different instants. However, once they are established the general behavior becomes again almost independent of inter‐prototype manufacturing imprecisions.

The “worst working condition” (P2) results in Figure 7f show, instead, how the preload, acting as a stabilization factor, leads to the shift of the folds formation region toward higher actuator pressure. Therefore, the corresponding peaks of standard deviation in Figure 7h result to be shifted for the same reason. Videos of the endocardial folds formation sequence (with and without preload) acquired with a laparoscope are available as (Video S3).

Figure 7e,f show also negative stroke volumes: this artifact is related to the preload application, when the volume of water stored in the ventricular chamber increases with respect to the designed value (113 mL), therefore, leading to a relative measure of the displaced volume of water. Although this artifact is not useful for the visualization of the real ejected stroke volume, it helps to better visualize the loading‐dependent behaviors. Instead, by taking into consideration the filling of the ventricle at 90 mmHg and therefore the absolute stroke volume value, computed as the difference between the total end‐diastolic and the end‐systolic volumes, it was possible to compare the ejection fraction phase of the two samples of soft robotic cardiac matrices (8‐actuators and 12‐actuators prototypes). Although it is worth mentioning that a 90 mmHg preload would never occur in a physiological situation, as visible in Figure 7d, the 12‐actuator sample were still able on average to reach in “worst working conditions” physiological stroke volumes (60.9 ± 2.5 mL, EF = 54%) by pressurizing the 12‐actuator sample at 120 kPa. Even better results were obtained in the “best working condition”, at 90 mmHg, where the absolute stroke volume reached was 77.2 ± 0.4 mL (EF = 68%).

## 5 Discussion

Recent advancements in soft robotics have significantly influenced material selection for healthcare applications, particularly in implantable cardiac systems. Soft materials offer promising alternatives for replicating the complex dynamics of the heartbeat. However, their non‐linear behavior and intricate structures often complicate modeling, making experimental validation a key aspect of the design process. The design in this study integrates two functional components: a bioinspired double‐helical myocardium mimicking muscular activity and a deformable endocardial layer that enhances volumetric reduction during the ejection phase. McKibben actuators were chosen for their ability to replicate muscular functionality. The optimal helix angle (63^**°**^) for the actuators correlated with the ejection fraction, consistent with values reported in the literature. This suggests that optimal design criteria from natural cardiac structures could be adapted to artificial systems, though achieving full biomimicry remains a challenge requiring further engineering development. To improve volumetric reduction, the stiffness of the endocardial material was optimized to induce stable folds. FEM simulations demonstrated that stiffer materials, like Smooth‐Sil 950, formed effective longitudinal folds at higher actuation pressures, enhancing ejection fractions and energy transmission efficiency. Preload application was shown to stabilize fold formation, shifting the fold formation region to higher actuation pressures and reducing variability. However, prototype‐to‐prototype variability due to manufacturing inconsistencies was observed, emphasizing the need for precise control over fold formation to ensure consistent performance.

The most effective prototype, comprising 12 aramid actuators in two counteracting layers and a Smooth‐Sil 950 endocardial layer, achieved an ejection fraction of 68% (77.2 ± 0.4 mL) under a 90 mmHg preload, meeting physiological benchmarks. These results represent an important step forward in replicating realistic cardiac function with soft robotics, demonstrating the feasibility of achieving physiological stroke volumes in artificial hearts. This study demonstrates the significant potential of soft robotics in addressing challenges in artificial cardiac systems. The successful achievement of physiological ejection fractions under preload conditions marks a critical milestone for the development of bioinspired total artificial hearts. Future research will focus on optimizing folding patterns, actuator configurations, and refining manufacturing processes. Dynamic testing under physiological conditions will be essential for improving prototype reliability and performance. These efforts will contribute to the development of more advanced bioinspired soft robotic technologies for total artificial heart applications, offering a promising path for the next generation of cardiac devices.

## Author Contributions

The author takes full responsibility for this article.

## Disclosure

Table of contents: A bioinspired design of a soft robotic artificial cardiac wall: the action of a double helical myocardium is coupled with an engineered passive and deformable endocardial layer. Experimental tests report an ejection fraction of 68%, i.e., 77.2 ± 0.4 mL against 90 mmHg, which satisfies physiological requirements. This work demonstrates how engineered bioinspiration might be the key to future artificial cardiac pumps.

## Conflicts of Interest

The authors declare no conflicts of interest.

## Supporting information


Video S1.

Appendix S1.

## Data Availability

The authors have nothing to report.
