# Supplementary material for: Design of a Soft Robotic Artificial Cardiac Wall
Source: Artif Organs. 2025 Mar 12;49(8):1265–76. doi: 10.1111/aor.14978 (PMC12269355; doi:10.1111/aor.14978)
Supplement: Supplementary file 2 — Appendix S1. [file AOR-49-1265-s001.zip › aor14978-sup-0009-Supplementary_Figure_3.pdf]

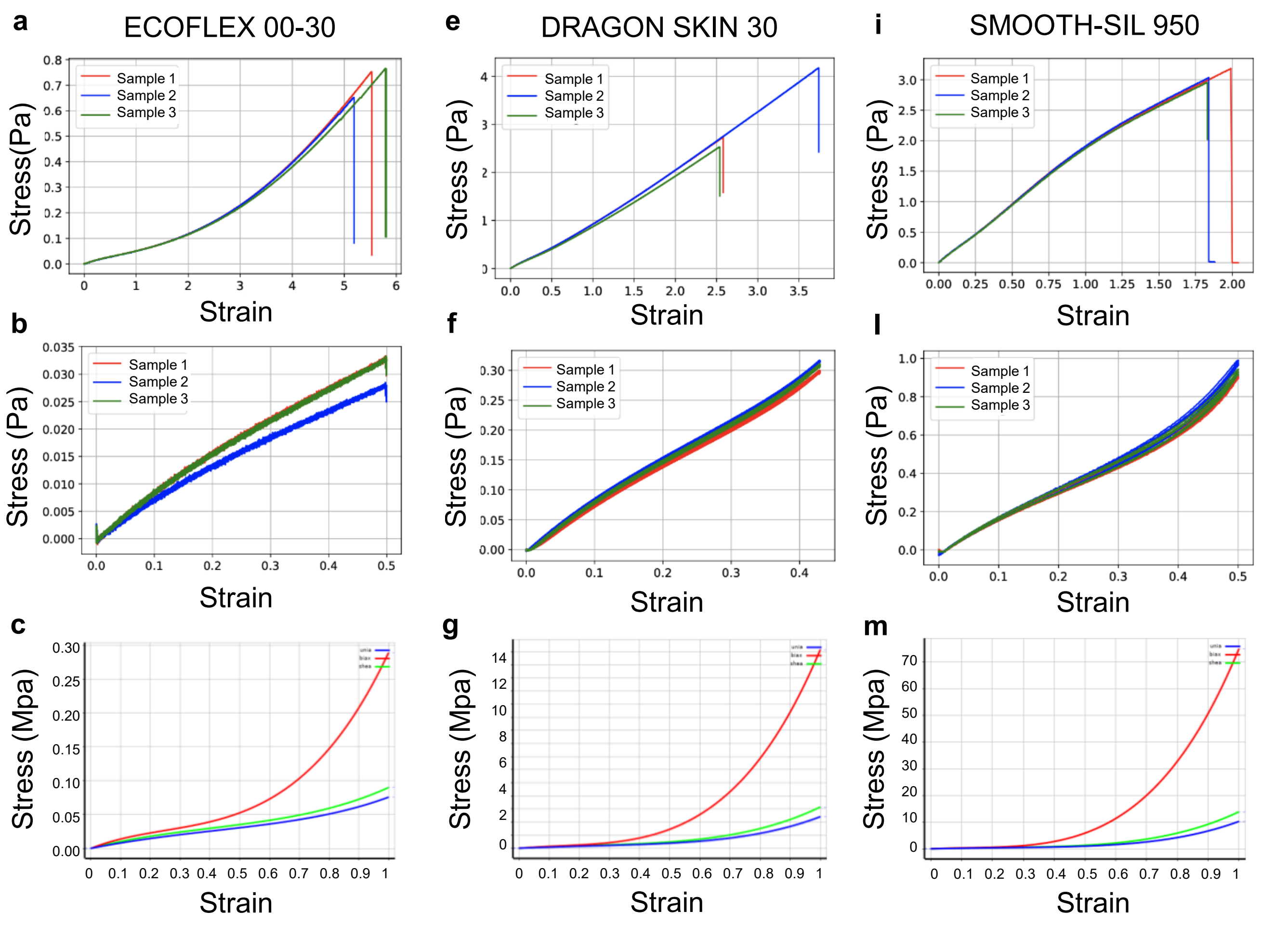

d Yeoh 3° Order E30

C10	0.35944 MPa
C20	-0.14221 MPa
C30	0.26123 Mpa
D1=D2=D3	0

h Yeoh 3° Order DS30

C10	0.1486 MPa
C20	-0.016429 MPa
C30	0.050278 MPa
D1=D2=D3	0

n Yeoh 3° Order SS950

C10	0.01458 MPa
C20	-0.00089 MPa
C30	0.00088 MPa
D1=D2=D3	0
