# Supplementary material for: Design of a Soft Robotic Artificial Cardiac Wall
Source: Artif Organs. 2025 Mar 12;49(8):1265–76. doi: 10.1111/aor.14978 (PMC12269355; doi:10.1111/aor.14978)
Supplement: Supplementary file 2 — Appendix S1. [file AOR-49-1265-s001.zip › aor14978-sup-0013-Supinfo.pdf]

Design of a Soft Robotic Artificial Cardiac Wall

*Debora Zrinscak Claudia M. De Chirico Lucrezia Lorenzon Fabiola Coluccia Mauro De Luca Martina Maselli Jolanda Kluin Johannes T.B. Overvelde Matteo Cianchetti**

D. Zrinscak, C. M. De Chirico, L. Lorenzon, F. Coluccia, M. De Luca, M. Maselli, M. Cianchetti
The BioRobotics Insitute and Department of Excellence of Robotics & AI, Scuola Superiore Sant'Anna, Viale Rinaldo Piaggio 34, 56025 Pontedera, Italy
Email Address: matteo.cianchetti@santannapisa.it
J. Kluin
Department of Cardiothoracic Surgery, Thorax Center, Erasmus MC Rotterdam, Dr. Molewaterplein 40, 3015 GD Rotterdam, The Netherlands
J. T. B. Overvelde
AMOLF, Science Park 102, 1098 XG Amsterdam, The Netherlands

Keywords: *Soft Robotics, Artificial Cardiac Wall, McKibben Actuators, Cardiovascular Disease, Heart Failure*

1 Supplementary Information

1.1 McKibben Actuators Fabrication

Each actuator's inflatable chamber was fabricated with an internal diameter of 3 mm and a wall thickness of 1 mm using Ecoflex 00-30 cured in custom 3D-printed molds at 60 °C for 1 h. Various braided sleeves were utilized, including thermo-formed polyester sleeves processed to achieve a cylindrical shape. The latter sleeving material was tested both with single and triple intertwining threads. The actuators were then roll-coated, thanks to a custom setup capable of processing up to 12 actuators simultaneously. Actuators were then cut to 70 mm for characterization purposes, and Smooth-Sil 950 (Smooth-On Inc., Macungie, PA) end-fittings were integrated via immersion bonding. Polyurethane tubes, glue (Sil-Poxy, Smooth-On Inc., Macungie, PA) and ties ensured air-tight connections. For myocardial applications, actuator lengths were adjusted to 185 mm and 210 mm for inner and outer myocardial layers, respectively. **Supplementary Figure 1** shows the molds and systems employed for the development of McKibben actuators. The elastomeric chambers were manufactured with two 3D printed mold elements, secured with screws to guarantee correct alignment. Each side presents two cylindrical grooves in order to produce two chambers per mold. The inner cavity of the chamber is manufactured by inserting a brass rod into the mold, see **Supplementary Figure 1a**. Once the mold was assembled, we slowly injected the silicone mixture through the injection holes. To prevent air bubbles, venting holes were placed at the top of the mold. After curing, the screws were unfastened, the sides were opened, and the rods were removed. We finished the elastomeric chambers by removing the lateral excess, visible in **Supplementary Figure 1b**, and, finally, we removed them from the rods. The main quotes of the molds are visible in **Supplementary Figure 1c**. The elastomeric chambers were then mounted onto the roll-coating setup in **Supplementary Figure 1d**. The silicone mixture was injected into the selected mesh and then slipped onto the chambers. A mold as the one in **Supplementary Figure 1e**, was filled with silicone and slowly approached the rotating setup. The rotational speed was adjusted to prevent the uncured material from dripping. At the end of the cure time the system was turned off and after four hours, the body of the actuators could be removed.

1.2 McKibben Actuators Testing

Three samples of each type of actuator underwent testing. For unloaded pressure-displacement tests, actuators were pressurized incrementally (from 15 kPa to burst, with 5 kPa steps) using a compressor (Leonardo, FIAC, Italy), a proportional pressure regulator (K8P-0-D522-0, CAMOZZI, Italy) and a pressure sensor (SWCN-P10-P3-2, CAMOZZI, Brescia, Italy). Data acquisition was managed through MATLAB (MathWorks), and axial contraction and radial expansion were evaluated via video analysis with

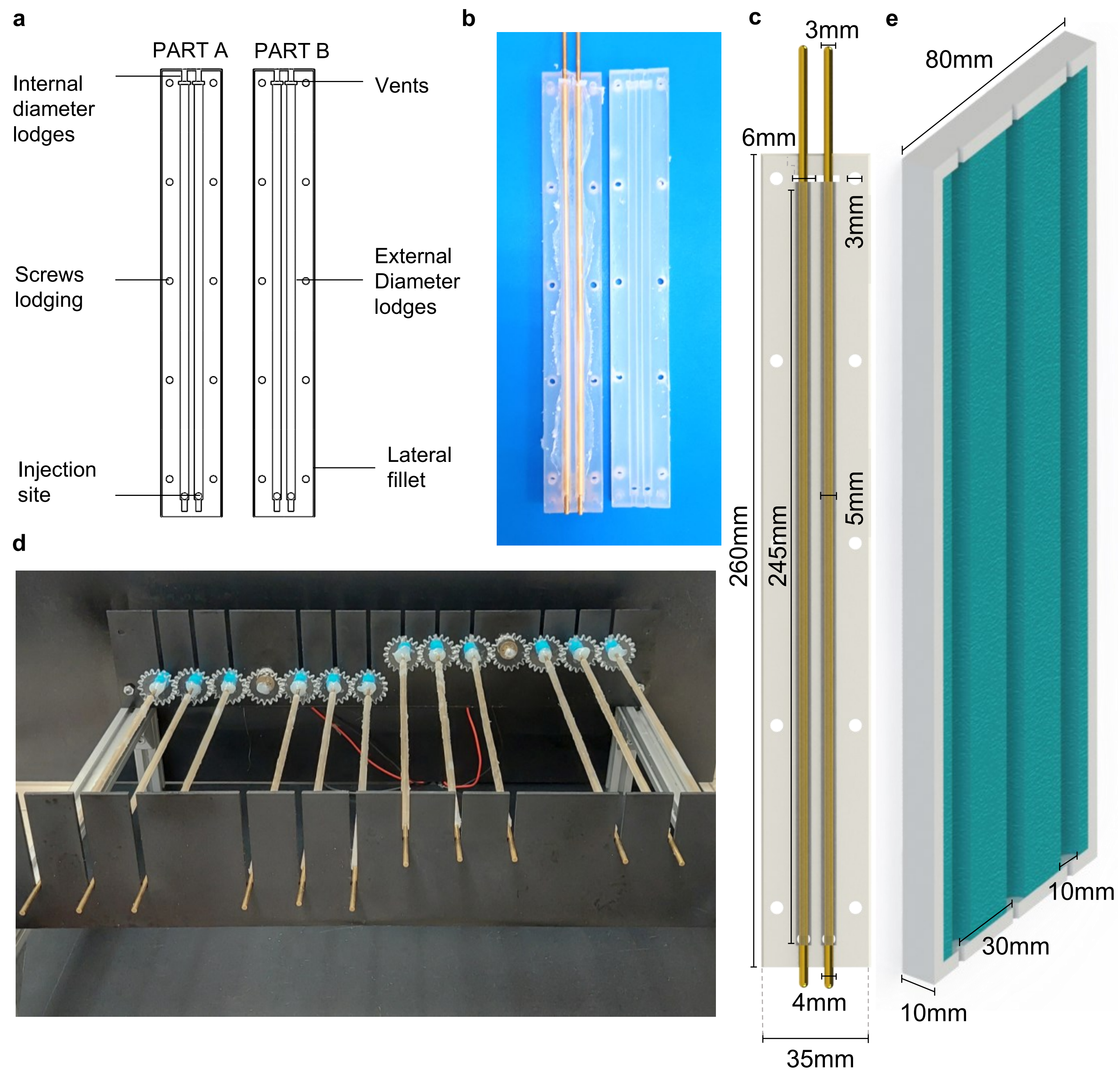

Supplementary Figure 1: McKibben actuators molds and fabrication setups. **a** CAD of the elastomeric chamber mold with description, **b** image of the cast elastomeric chambers **c** rendering of the mold with quotes. **d** Roll-coating setup. **e** Batch employed for the roll-coating of the McKibben actuators with quotes.

motion-tracking software (Tracker, Physlets.org). MATLAB scripts processed mean pressure-displacement behavior and bursting pressures. The loaded behavior of the actuators was instead evaluated by measuring the length and diameter of each sample mounted on a complete artificial cardiac wall prototype. Force-displacement tests utilized a tensile testing machine (INSTRON 5965, USA) combined with the same setup employed in pressure-displacement tests with a pressurization step of 20 kPa. The actuator was clamped, by tightening the end fittings to avoid slipping, and the machine displacement was set to zero. The actuator was then pressurized and the crossbar moved to reach a null force value. The INSTRON machine was then controlled in displacement until zero-displacement was reached, and the force values were recorded. Data were averaged using custom MATLAB code.
Summarizing information about the pressure-displacement tests is available in **Supplementary Table**

1. The best-performing samples in this type of test, taking into consideration both the contraction ratio and the average bursting pressure, are identified. **Supplementary Table 2** summarizes the force-displacement test results, and the two actuators able to develop the highest force at 100 kPa are identified. The samples characterized by an aramid sleeve, resulted in being the most promising ones for the intended application, as they performed well in both cases. The mean elastic modulus, successively employed in the FEM simulation, was computed by averaging the elastic moduli obtained at the different tested actuating pressures. In particular, each Young's Modulus was computed as the ratio between stress and strain, obtained with the force-displacement tests, as $\frac{FL_0}{A(L_d-L_0)}$, where F is the maximum force at a certain pressure level, L_0 is the rest length, L_d is the maximum deformed length and A the cross-section. **Supplementary Figure 2** displays all the mean results of the force-displacement tests per each McKibben sample.

Supplementary Table 1: Results of pressure-displacement tests. The table displays the acronym of each typology of actuator, the contraction percentage at 100 kPa, the average bursting pressure and the initial braiding angle. The best-performing actuator typologies are E30-A-E30 and E30-I-E30.

Acronym	% Contraction @100 kPa	Average Bursting Pressure (kPa)	Braiding Angle (°)
E30-S-E30	27.2	160	20
E30-T-E30	25.6	190	20
E30-S-D10	18.9	300	20
E30-A-E30	22.4	270	20
E30-C-E30	15.3	430	25
E30-I-E30	23.1	380	25
E30-SI-E30	19.7	250	25

Supplementary Table 2: Results of force-displacement tests. The table displays the acronym of the actuator typology, the percentual contraction, the mean force value at 100 kPa, the mean elastic modulus and the elastic modulus at 100 kPa. The best-performing actuators typologies are E30-A-E30 and E30-SI-E30.

Acronym	% Contraction @100 kPa	Force (N) @100 kPa	Mean Elastic Modulus (MPa)	E(MPa) @100 kPa
E30-S-E30	21.7	23	2.19	2.66
E30-T-E30	21.3	23	2.44	2.56
E30-S-D10	16.7	19	3.64	3.14
E30-A-E30	22.5	34	3.99	2.93
E30-C-E30	12.7	20	4.93	3.80
E30-I-E30	18.6	23	2.65	2.58
E30-SI-E30	20.5	30	4.26	3.68

1.3 Finite Element Modeling

To model the endocardial material properties and simulate them in the ANSYS 2021 R1 Workbench platform, we characterized the three selected materials (Ecoflex 00-30, Dragon Skin 30, Smooth-Sil 950) following ASTM and ISO standards. Three dogbone samples per material were fabricated. They all had a surface of 12 mm^2, a testing length of 350 mm and a thickness of 2 mm. Two types of tests were performed employing a universal tensile testing machine (INSTRON 5965, Norwood,MA,USA). The first one, a rupture test, was used to define the safest testing range. The second one, a cyclic test, repeated 5 times at 50% their rest length, was employed to determine the strain-stress curves of the materials. In both cases, the testing speed was set to 50 mm/min. The obtained results are summarized, respectively, in the first and second row of **Supplementary Figure 3**. Ecoflex 00-30 samples broke around a strain of 550%, Dragon Skin 30 ones around 300% and Smooth-Sil 950 ones around 180%. A Yeoh third-order model was used to model the behavior of the tested hyperelastic materials. We fitted in ANSYS

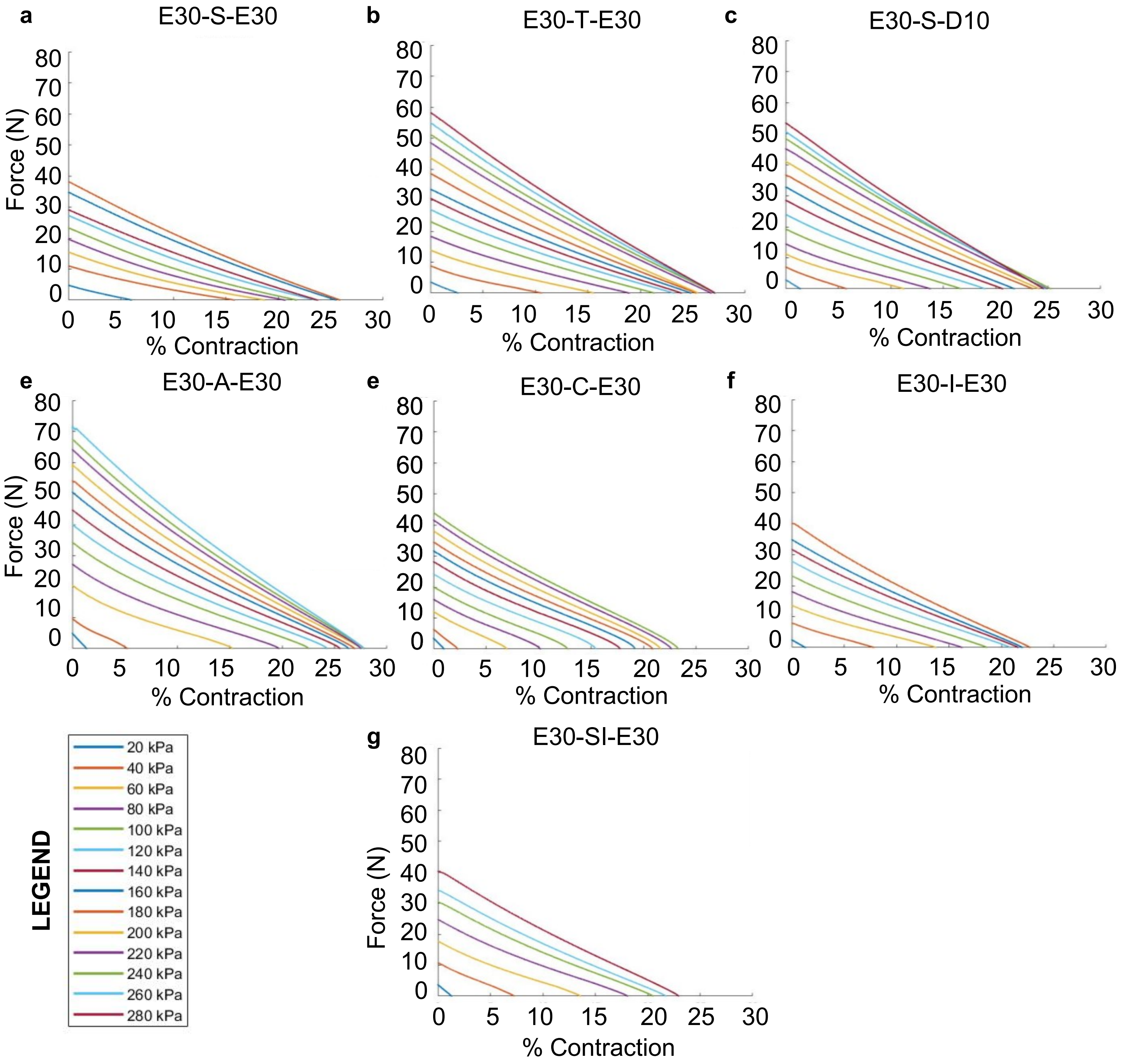

Supplementary Figure 2: Results of the McKibben actuators force-contraction characterization. **a** E30-S-E30 Single polyester thread (Ecoflex 00-30 coating, if not differently specified), **b** E30-T-E30 triple polyester thread, **c** E30-S-D10 Single polyester thread (Dragon Skin 10 coating), **d** E30-A-E30 aramid mesh, **e** E30-C-E30 carbon fiber mesh, **f** E30-I-E30 innegra braided sleeve **g** E30-SI-E30 silane mesh.

the cyclic behavior obtaining the curves visible in the third row of **Supplementary Figure 3**, and the parameters in the fourth one.
As specified in the Experimental Section 'FEM model', the behavior of the McKibben actuators was simulated as suggested by Roche et al. [?]. Each portion of the actuator was modeled as a material characterized by the experimentally obtained Young's Modulus, a Poisson ratio of 0.35 [?], and radial and axial coefficients of thermal expansion determined analytically by mapping the experimental radial and axial strain of the tested McKibben actuators. By imposing a thermal expansion in ANSYS is therefore possible at each load step to compute an increment of thermal deformation with respect to the initial dimensions and temperature values. The temperature-dependent orthotropic secant thermal expansion

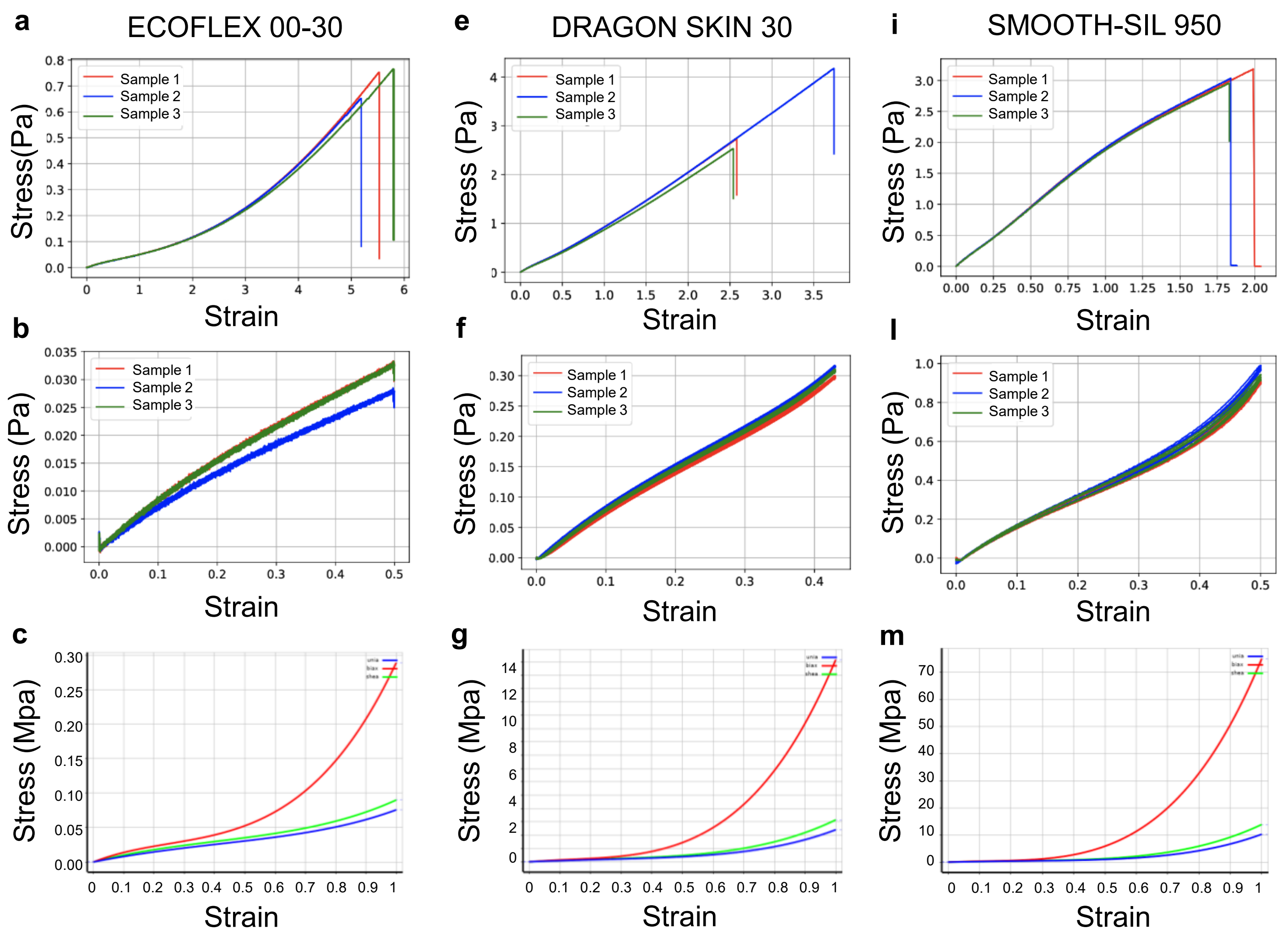

d Yeoh 3° Order E30

C10	0.35944 MPa
C20	-0.14221 MPa
C30	0.26123 Mpa
D1=D2=D3	0

h Yeoh 3° Order DS30

C10	0.1486 MPa
C20	-0.016429 MPa
C30	0.050278 MPa
D1=D2=D3	0

n Yeoh 3° Order SS950

C10	0.01458 MPa
C20	-0.00089 MPa
C30	0.00088 MPa
D1=D2=D3	0

Supplementary Figure 3: Material characterization tests and results. **a-d** Ecoflex 00-30 material characterization and modeled behavior: **a** rupture tests to determine the working range, **b** cyclic tests to evaluate the stress-strain curves **c** Yeoh third-order model fitting curve **d** relative parameters. **e-h** Dragon Skin 30 material characterization and modeled behavior with parameters, **i-n** Smooth-Sil 950 material characterization and modeled behavior with parameters.

coefficients were, thus, determined by applying the following equation:

$$\alpha(T) = \frac{ln(\alpha(T - T_0) + 1)}{T - T_0} \tag{1}$$

Where α is the temperature-dependent coefficient of thermal expansion, T is the considered temperature, and T_0 is the reference temperature. This parameter can be linked to the experimental behavior of the actuator through the strain value ϵ evaluated in the axial or radial direction as follows:

$$\begin{cases} \alpha_a = \frac{\epsilon_a}{\Delta T} \\ \alpha_r = \frac{\epsilon_r}{\Delta T} \end{cases} \tag{2}$$

The experimental values of $\epsilon(P)$ were referred to as the McKibben actuator behavior in a selected state, loaded or unloaded. The thermal load applied within the simulation environment is the following:

$$\Delta(T) = (T - T_0) = 122 - 22 = 100°C \tag{3}$$

The method was first tested by simulating a single McKibben actuator with Smooth-Sil 950 end-fittings, and, successively, applied to each portion of the helically wrapped actuator of the artificial myocardial layers.
For what concerns the simulation settings, we set a static structural model. Due to the presence of hyperelastic materials, we activated the large deflection mode. We employed tetrahedral quadratic elements, and we kept the default element size. The element curvature was varied in a range of 58° and 62° to help the convergence of the model. The initial substeps were set to 100, while the minimum and maximum to 10 and 1000, to increment the load slowly and prevent excessive distortion of the elements. Due to the model instabilities, a stabilization parameter was used to help the convergence, introducing a constant energy dissipation ratio equal to 1. We always verified that the stabilization energy was much lower than the deformation energy value. A summary of mesh statistics for different simulated materials is reported in **Supplementary Table 3**.

**Material**	**Max AR**			**Max Jacobian Ratio**			**Max Skewness**		
	Warning (5)	Failed (1000)	Avg	Warning (0.05)	Failed (0.025)	Avg	Warning (0.9)	Failed (0.999)	Avg
Ecoflex 00-30 & Dragon Skin 00-30	1.287%	0%	2.229%	0%	0%	0.746	0.473%	0%	0.425
Smooth-Sil 950	18.055%	0%	3.129%	0%	0%	0.762	3.645%	0%	0.53

Supplementary Table 3: Mesh statistics summary for different materials.

1.4 Prototype Fabrication

The fabrication of the prototype started by casting the ventricular chamber, with the molds depicted in **Supplementary Figure 4a and b**. The first one served to obtain a semi-ellipsoidal shell of desired dimensions. While the dimensions of the inner part, also referred to as the core, were kept the same dimensions (semi-major axis of 60 mm and semi-minor axis of 30 mm), the external ones, varied depending on the thickness we needed to obtain (3 mm for the Ecoflex 00-30 and Dragon Skin 30 endocardia, and 1.5 mm for the Smooth-Sil 950 one). First, the external elements of the molds were correctly aligned with screws. Then the silicone mixture was prepared. While degassing, a mold release product was sprayed and brushed onto all the mold elements (Universal Mold Release 200 or Universal Mold Release, Smooth-On Inc.). When ready, the mixture was poured into the assembled bottom until approximately one-third of its height. At this point, the core was pushed slowly inside the bottom to allow even distribution of the elastomeric mixture. Finally, the two parts aligned by clicking the core into the bottom, and the silicone was cured in an oven for the required amount of time at 60°C. The second mold, shown in **Supplementary Figure 4b**, was employed to manufacture the outlet tubes. In this case, the mold is composed of two bottom elements, one top circular element and two cores. As previously, the two bottom elements were aligned with screws, and the top element was secured with some hot glue to prevent unwanted displacements. The mold release agent was sprayed and brushed onto the elements, while the already prepared silicone mixture was being degassed. In this case, the cores were placed into the assembled mold, and the silicone was injected with a syringe from the top. The model was cured in an oven at 60°C. Usually, these two elements were fabricated simultaneously, and a small amount of uncured silicone was preserved at -4°, until they were fully cured. Once the semi-ellipsoidal shell and the outflow

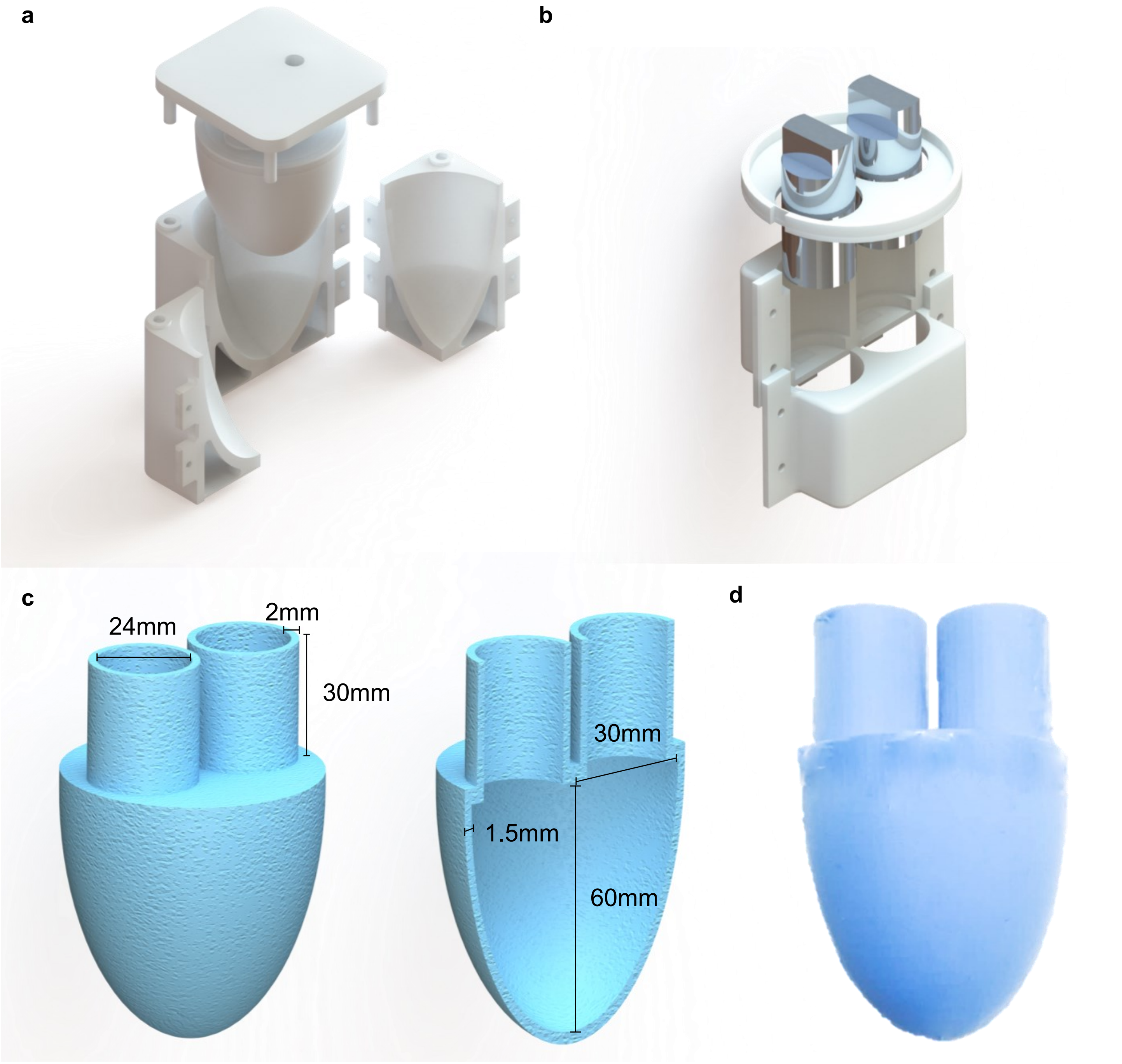

Supplementary Figure 4: Molds employed to fabricate the endocardial layer. **a** CAD the ventricular shell mold: the bottom is divided into four elements, while the core is a single element. **b** Multi-element mold for the realization of the outflow tubes. **c** Renderings with quotes of an assembled Smooth-Sil 950 ventricle. **d** Image of a cast Smooth-Sil 950 endocardial layer.

tubes were ready, they were demolded and finished with scissors to remove the excess silicone burrs. Finally, the semi-ellipsoidal shell was placed into its bottom mold and bonded to the top with the uncured silicone. The renderings of the ventricle and an image of the casted prototype are visible in **Supplementary Figure 4c and d**.
To determine the relative position of the helices with respect to each other and the ventricular shell, we draw schematics as the one depicted in **Supplementary Figure 5a**. Renderings of the assembled, 12-actuator and 8-actuator prototypes are visible in **Supplementary Figure 5b-c**, respectively. After determining the axial coordinates of each helix in MATLAB, we used the data to sweep-cut the Solidworks parts of top **Supplementary Figure 5d-e** and bottom plates **Supplementary Figure 5f-g**.

The plates served to keep the actuators in the correct orientation and prevent the bulging of the soft endocardial top, upon actuators' pressurization. Moreover, they were used to interface the endocardial shell with the double helical myocardium. The latter was obtained by casting a thin elastomeric layer on top of the correctly positioned actuators, as visible in **Supplementary Figure 5h-i**. The renderings of the molds are provided with quotes. The semi-ellipsoid is drawn in order to obtain a 0.50 mm membrane, and the grooves are cut slightly larger to better accommodate the bent actuator. On the base of the dimension of the final mold surfaces **Supplementary Figure 5l-m**, we were able to compute the necessary volume to cast the connective elastomeric membranes. The prototype was assembled by positioning the outer layer on the bottom plate, then inserting the inner layer, and finally gluing the endocardial shell to it using Multifiss adhesive (PROCHIMICA, Italy). Successively the outflow tubes were inserted into the top plate, followed by the inner and the outer membrane actuators, paying attention to the correct relative orientation of the two plates. Finally, the bottom ends of the actuators were filled with glue and sealed with a plastic tie. Tubes were placed into the top ends, and two plastic ties per actuator were employed, one on top of the plate, and the other underneath it, to block the position. Glue was used to further seal the actuator and the tube.

1.5 Experimental tests with laparoscope

To study the endocardial deformation we conducted tests with a laparoscopic instrument (Hopkins II 26003BA, Karl Storz). These tests aimed at qualitatively evaluating the formation of endocardial folds when activating the soft robotic cardiac wall at different frequencies, actuator pressures and ventricular loading. Videos are available as Supporting Information. A schematic view of the setup is visible in **Supplementary Figure 6a**. The instrumentation employed is the same as in the ejection phase test - exception made for the dimensions of the plexiglass tube used as a connection with the physiological pressure simulator circuit. The substituting tube had a slightly larger diameter (24 mm vs 18 mm) and a longer length (1200 mm vs 600 mm). This change allowed to contain a higher water volume during actuation, and, therefore use the second outflow tube as inlet for the imaging instrument. The laparoscope was inserted in a custom-made fitting, which served as an interface with the outflow tubes, and kept the visualization instrument at an angle to constantly and correctly acquire videos and images of the center. We performed two typologies of tests. The first one aimed at studying the timing of the formation of folds, upon increasing pressure in the actuators, without any ventricular loading. **Supplementary Figure 6b-c** display the tested prototype in diastolic and systolic conditions (0 kPa and 120 kPa respectively). The internal formation of folds at different actuating pressures is visible in **Supplementary Figure 6d-f** (60, 90 and 120 kPa respectively). The second one, instead, verified the formation of the folds in dynamic conditions, by actuating the prototype with a frequency of 0.5 Hz at different maximum pressures, and different loading conditions. In general, we selected an actuating pressure and frequency and set, if required, a preload pressure in the ventricle. After applying the preload, the actuation started and the formation of folds was observed. It is important to underline, that upon actuation the measured ventricular pressure increased depending on the developed constrictive force. Although just a few quantitative data were extracted, mostly related to the ventricular loading ramp, it was possible to make interesting considerations. On the one hand, it was possible to understand that the folds timing is strongly influenced by the manufacturing. This well explained the inter-prototype variability visible in the ejection phase results. Moreover, the folds typology was strongly influenced by the ventricular loading: a higher preload led to smaller folds and, thus, to a lower ejected volume. On the other hand, with these considerations, we can hypothesize future improvements of the presented device. By standardizing the manufacturing procedure and designing strategic weak points, we could improve the final systolic volume and reduce the inter-prototype variability, building more reliable and performing devices.

1.6 List of Supplementary Videos

Supplementary Video 1 - Concept: the video summarizes the motivation, the design and the main results of the paper.

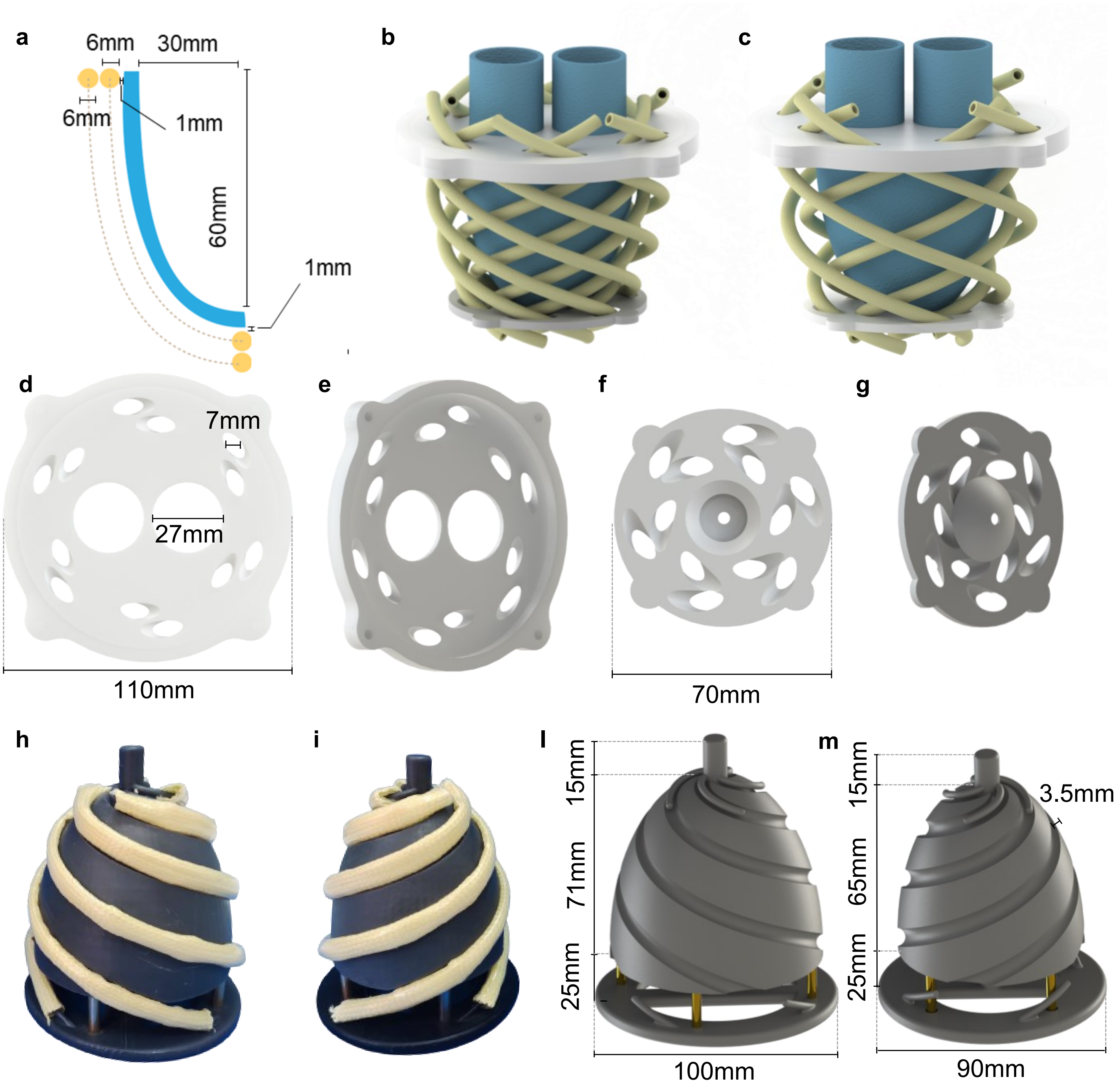

Supplementary Figure 5: Molds employed to fabricate and assemble the prototype. **a** Schematics on the base of which the actuator helices were drawn. **b** Rendering of the assembled 12-actuator prototype. **c** Rendering of the assembled 8-actuator prototype. **d-e** Renderings of the 12-actuator prototype top plate. **f-g** renderings of the 12-actuator prototype bottom plate. **h-i** Pictures of the 8-actuator 3D-printed mold with assembled actuators (outer and internal layer). **l-m** Rendering of the molds employed for the outer and inner myocardial layer fabrication.

Supplementary Video 2 - Experimental Tests: the video shows the main experimental setups and some examples of performed tests, underlining the main observations.
Supplementary Video 3 - Endocardial Folds: the video shows the experimental setup and the results of the qualitative study. It displays videos of the endocardial fold formation process.

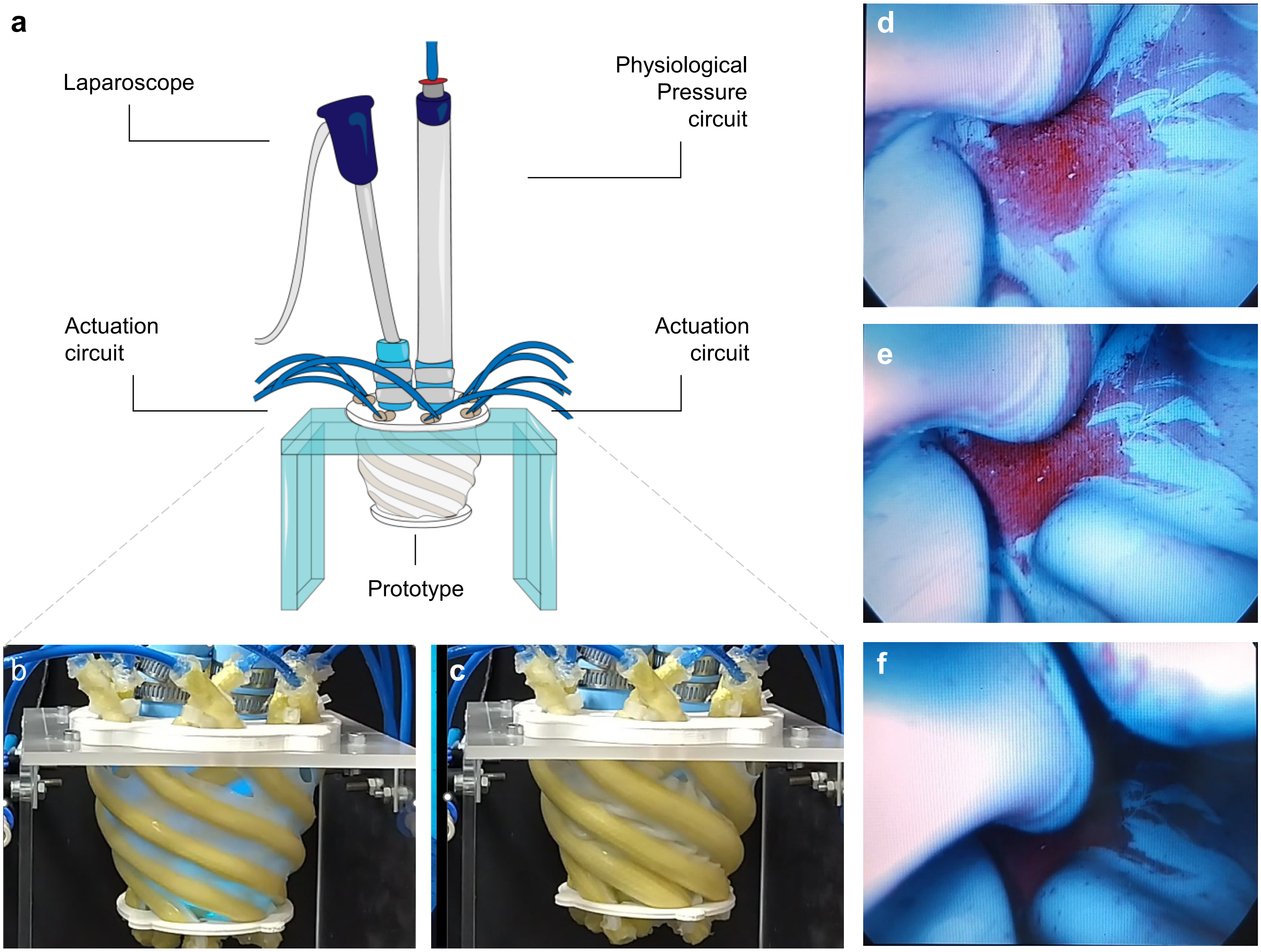

Supplementary Figure 6: Folding pattern observed with the laparoscopic tests. **a** Schematics of the testing setup. **b** Diastolic phase: actuators pressure at 0 kPa **c** Systolic phase: actuators pressure 120 kPa. **d-f** Ventricular folds at 60, 90 and 120 kPa, respectively, without ventricular loading, and at an actuation frequency of 0.5 Hz.
