# Supplementary figures and images for: Design of a Soft Robotic Artificial Cardiac Wall
Source: Artif Organs. 2025 Mar 12;49(8):1265–76. doi: 10.1111/aor.14978 (PMC12269355; doi:10.1111/aor.14978)

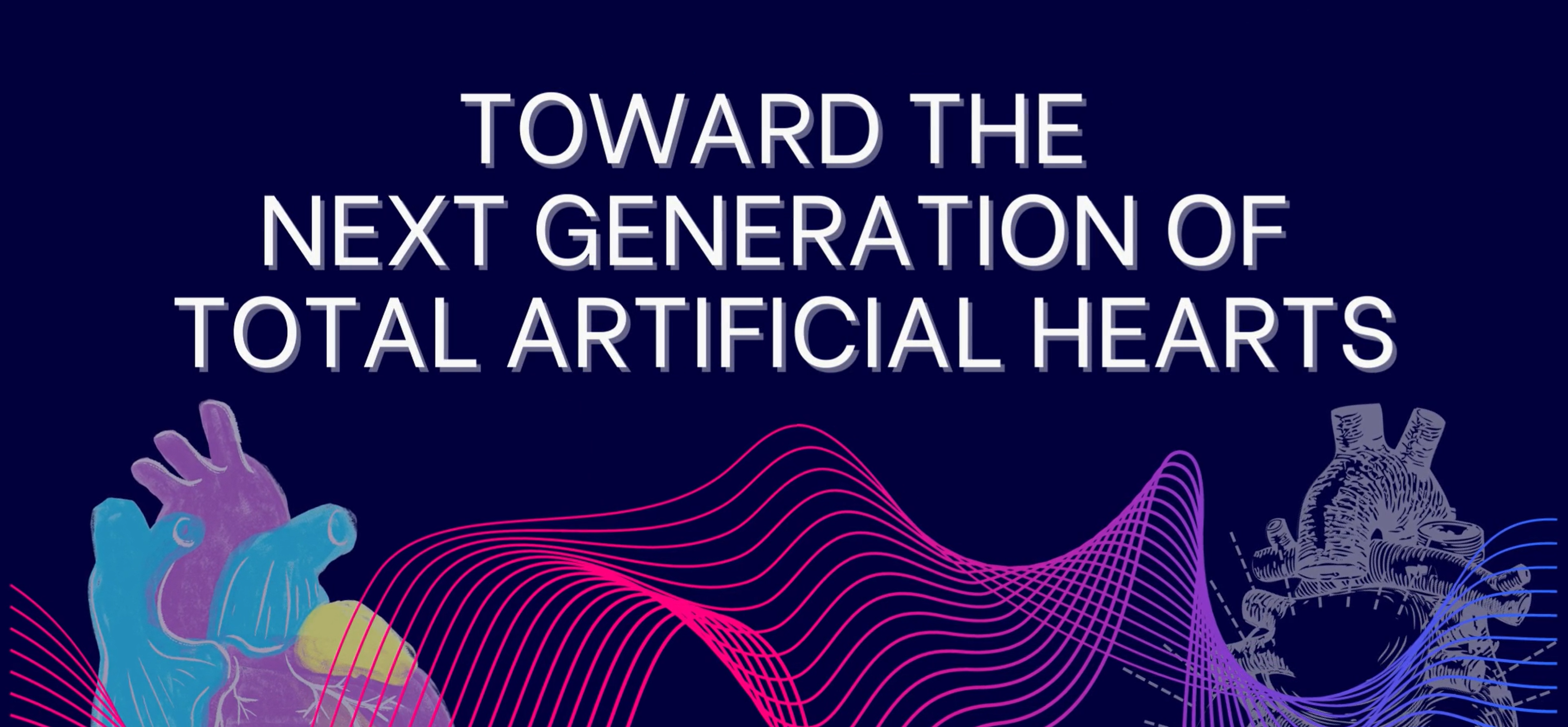

Supplement: Supplementary file 2 — Appendix S1. [file AOR-49-1265-s001.zip › aor14978-sup-0001-supinfo_placeholder_S1.png]

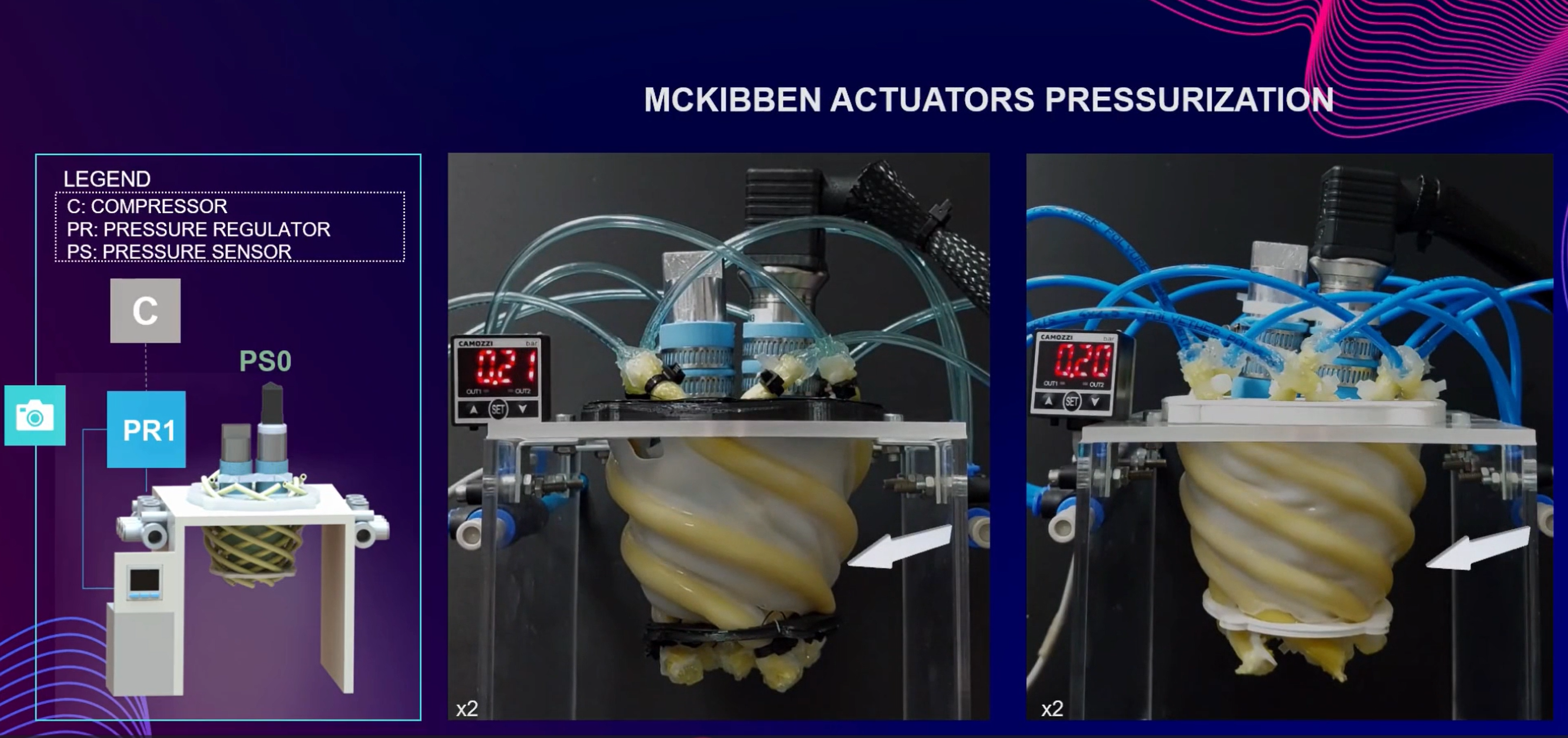

Supplement: Supplementary file 2 — Appendix S1. [file AOR-49-1265-s001.zip › aor14978-sup-0002-supinfo_placeholder_S2.png]

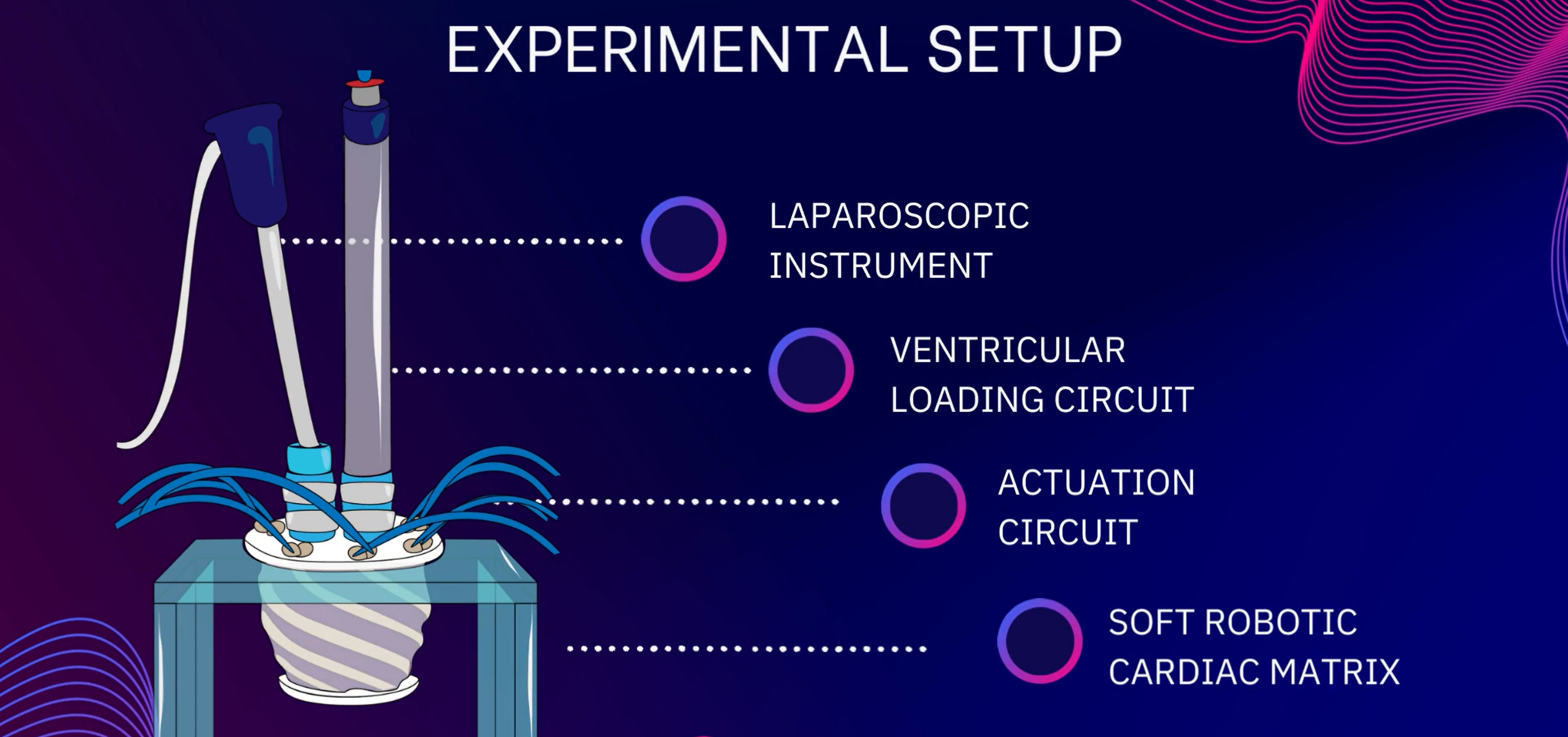

Supplement: Supplementary file 2 — Appendix S1. [file AOR-49-1265-s001.zip › aor14978-sup-0003-supinfo_placeholder_S3.png]

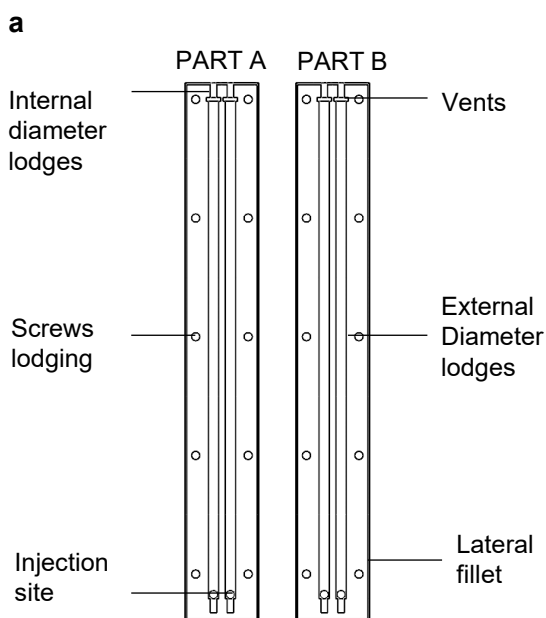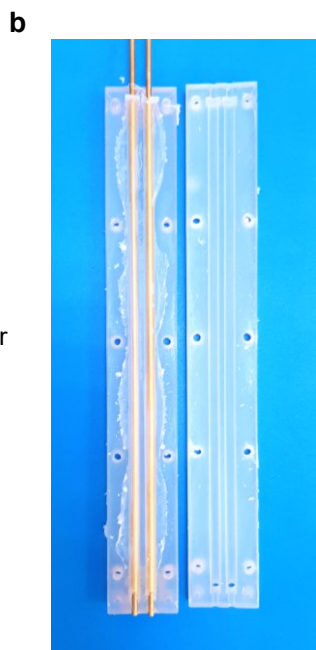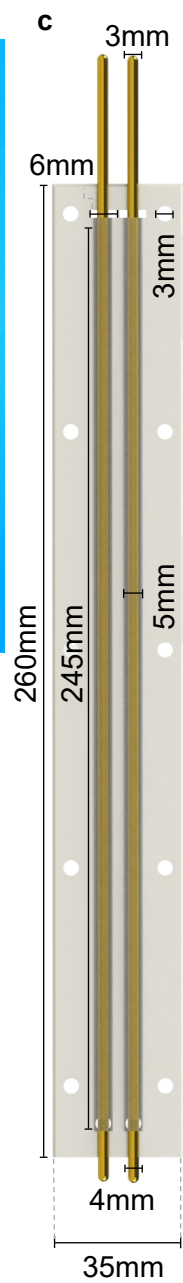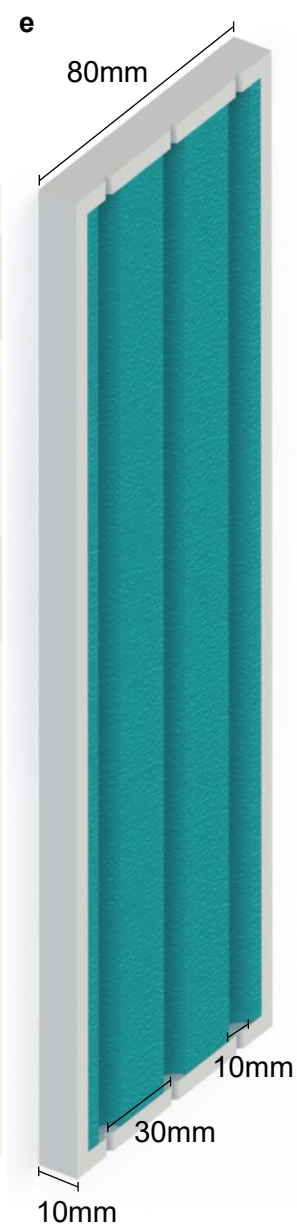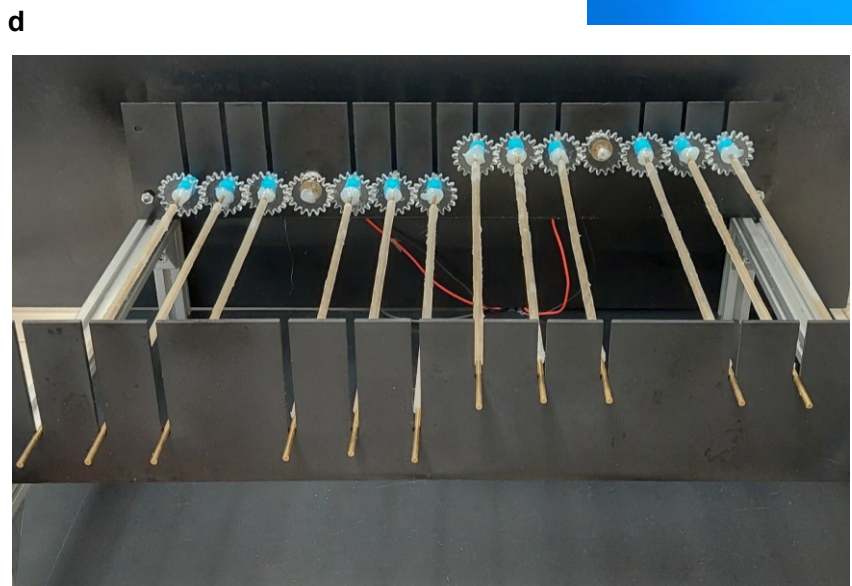

Supplement: Supplementary file 2 — Appendix S1. [file AOR-49-1265-s001.zip › aor14978-sup-0007-Supplementary_Figure_1.pdf]

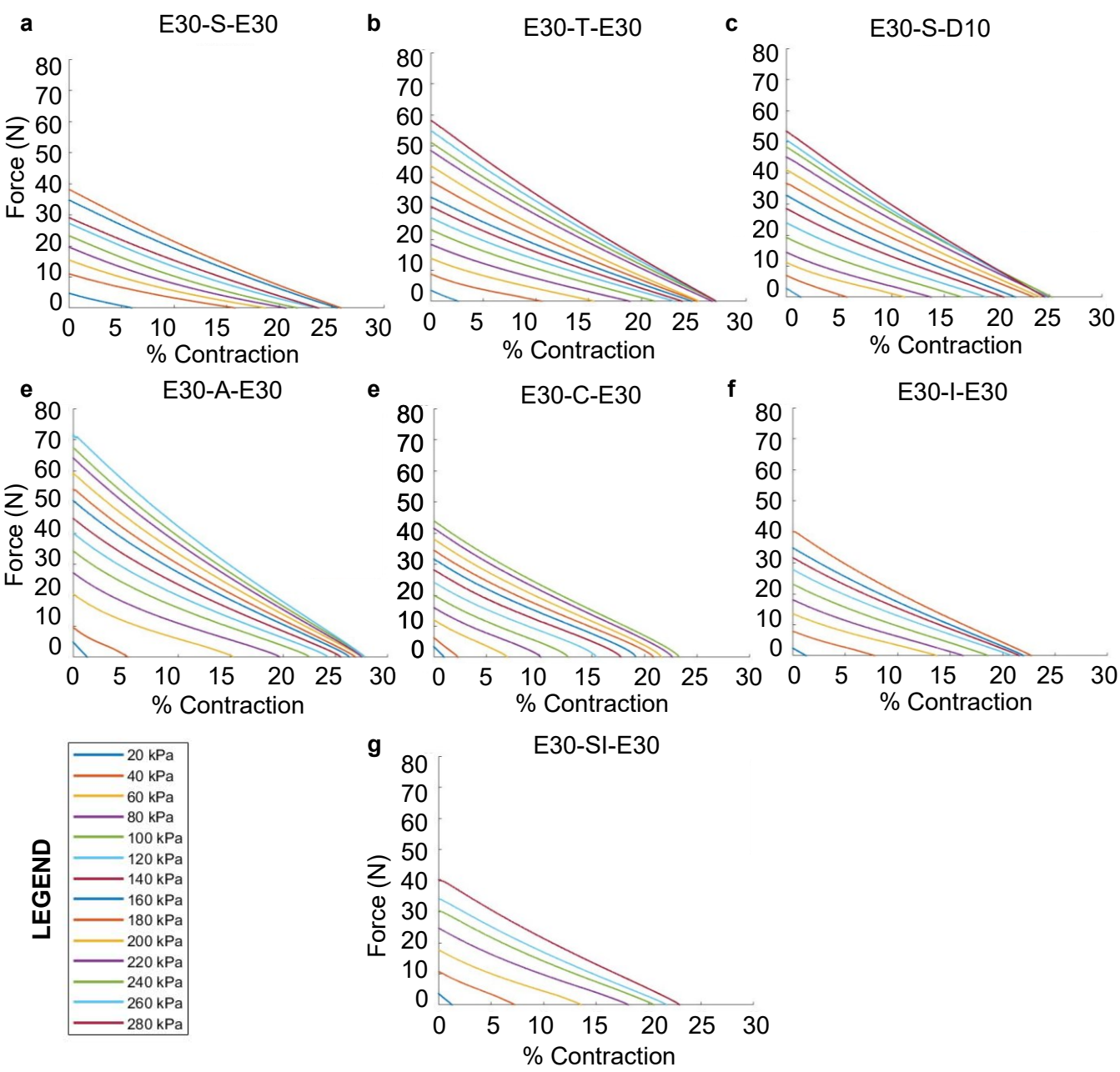

Supplement: Supplementary file 2 — Appendix S1. [file AOR-49-1265-s001.zip › aor14978-sup-0008-Supplementary_Figure_2.pdf]

**a**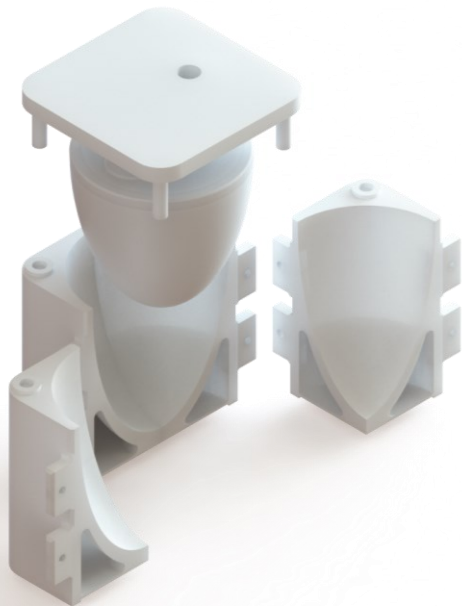**b**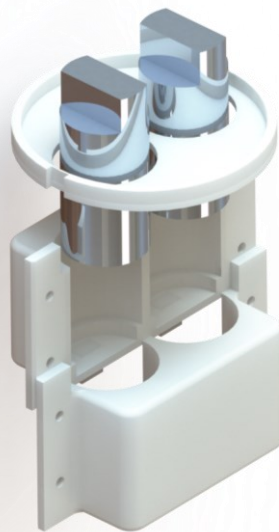**c**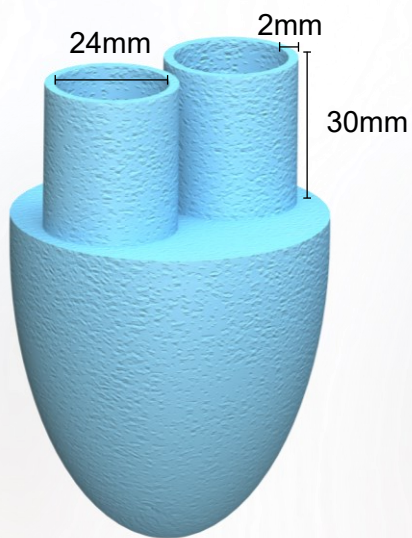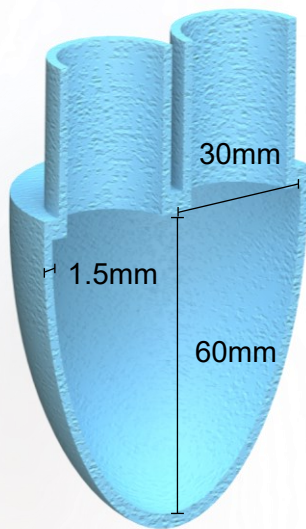**d**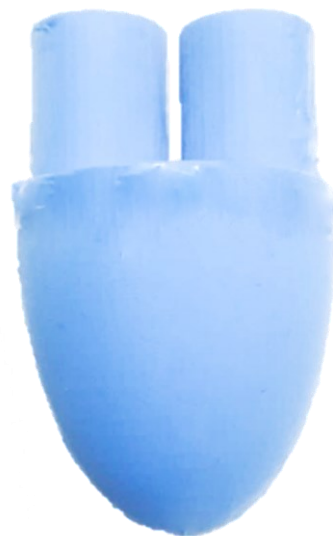

Supplement: Supplementary file 2 — Appendix S1. [file AOR-49-1265-s001.zip › aor14978-sup-0010-Supplementary_Figure_4.pdf]

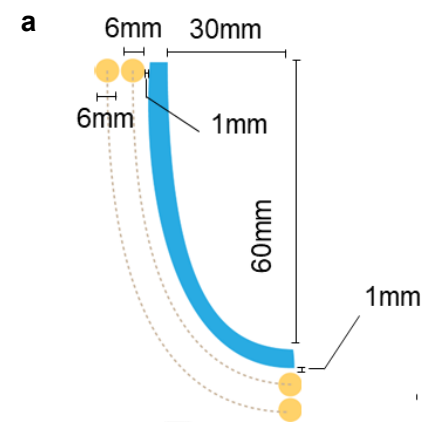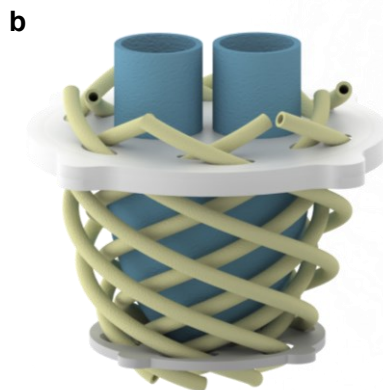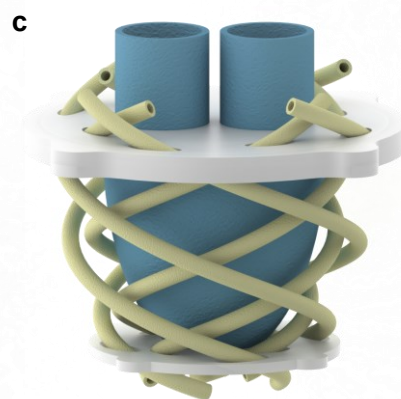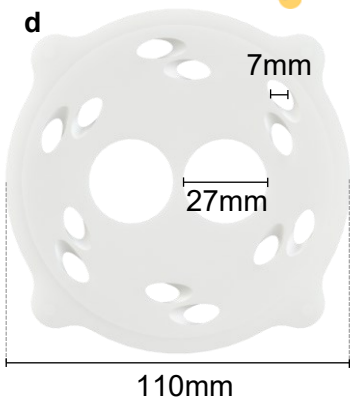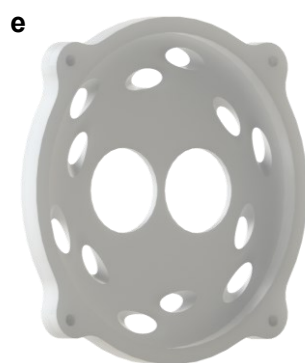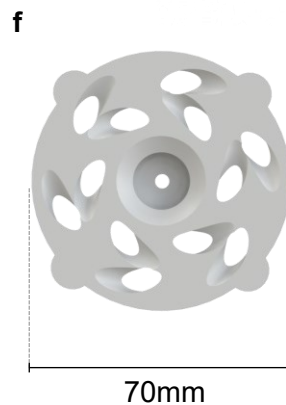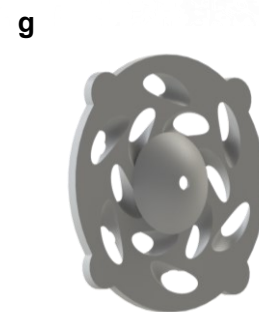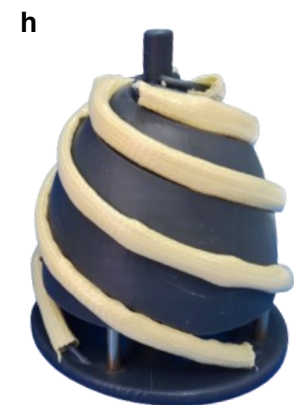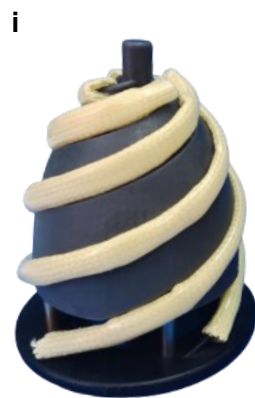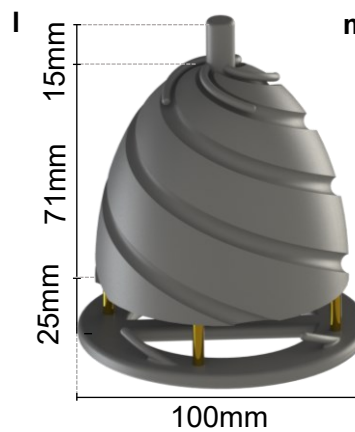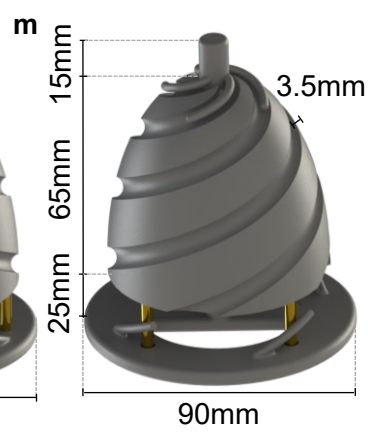

Supplement: Supplementary file 2 — Appendix S1. [file AOR-49-1265-s001.zip › aor14978-sup-0011-Supplementary_Figure_5.pdf]

**a**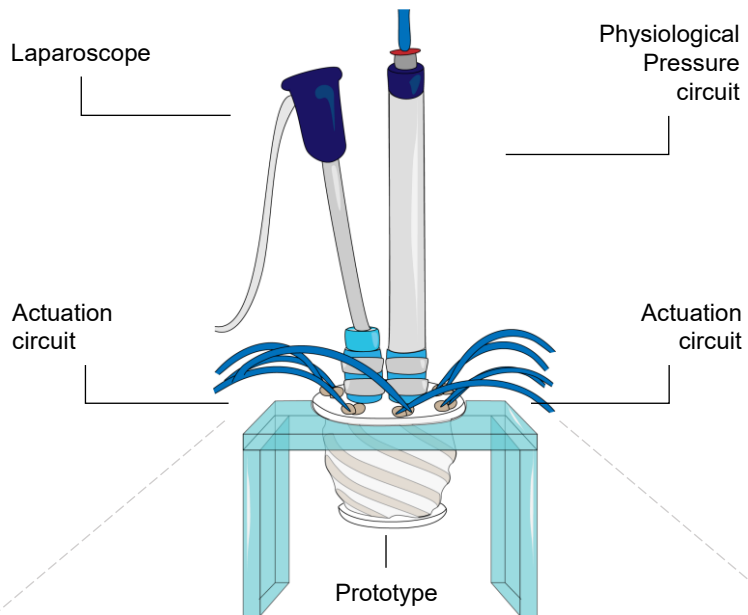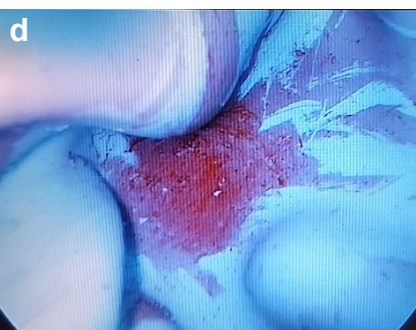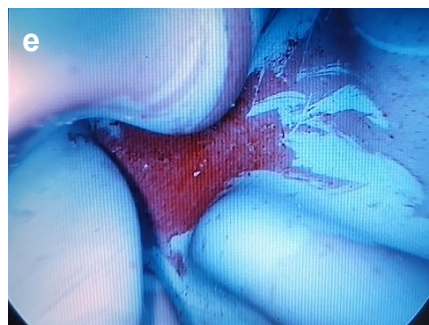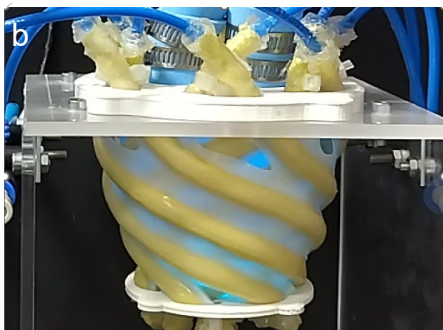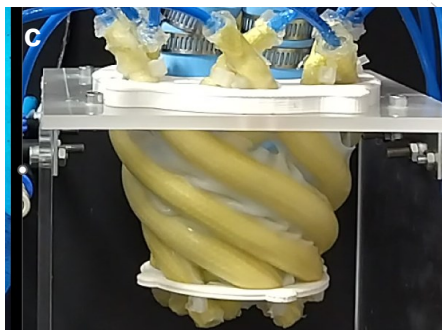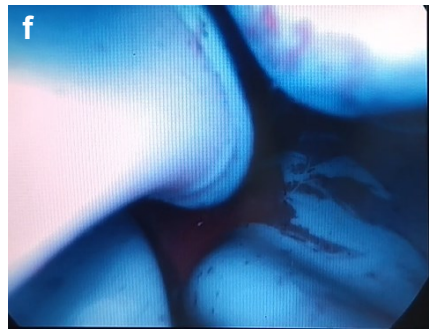

Supplement: Supplementary file 2 — Appendix S1. [file AOR-49-1265-s001.zip › aor14978-sup-0012-Supplementary_Figure_6.pdf]
